# Mendelian randomization and experimental IUGR reveal the adverse effect of low birth weight on lung structure and function

**DOI:** 10.1038/s41598-020-79245-7

**Published:** 2020-12-28

**Authors:** Celien Kuiper-Makris, Daniela Zanetti, Christina Vohlen, Luise Fahle, Marion Müller, Margarete Odenthal, Ursula Felderhoff-Müser, Jörg Dötsch, Miguel A. Alejandre Alcazar

**Affiliations:** 1 Translational Experimental Pediatrics-Experimental Pulmonology, Department of Pediatric and Adolescent Medicine, Faculty of Medicine and University Hospital Cologne, University of Cologne, Kerpener Strasse 62, 50937 Cologne, Germany; 2 Department of Pediatric and Adolescent Medicine, Faculty of Medicine and University Hospital Cologne, University of Cologne, Cologne, Germany; 3 Division of Cardiovascular Medicine, Department of Medicine, Stanford University School of Medicine, Stanford, CA USA; 4 Stanford Cardiovascular Institute, Stanford University, Stanford, CA USA; 5 Institute of Pathology, Faculty of Medicine and University Hospital Cologne, University of Cologne, Cologne, Germany; 6 Center for Molecular Medicine Cologne (CMMC), Faculty of Medicine and University Hospital Cologne, University of Cologne, Cologne, Germany; 7 Department of Paediatrics I, University Hospital Essen, University Duisburg-Essen, Essen, Germany; 8 Institute for Lung Health, Member of the German Centre for Lung Research (DZL), University of Giessen and Marburg Lung Centre (UGMLC), Giessen, Germany; 9 Cologne Excellence Cluster for Stress Responses in Ageing-Associated Diseases (CECAD), Cologne, Germany

**Keywords:** Respiratory tract diseases, Translational research, Paediatric research

## Abstract

Intrauterine growth restriction (IUGR) and low birth weigth (LBW) are risk factors for neonatal chronic lung disease. However, maternal and fetal genetic factors and the molecular mechanisms remain unclear. We investigated the relationship between LBW and lung function with Mendelian randomisation analyses and studied angiogenesis in a low protein diet rat model of IUGR. Our data indicate a possible association between LBW and reduced FEV1 (p = 5.69E−18, MR-PRESSO) and FVC **(**6.02E-22, MR-PRESSO). Complimentary, we demonstrated two-phased perinatal programming after IUGR. The intrauterine phase (embryonic day 21) is earmarked by a reduction of endothelial cell markers (e.g. CD31) as well as mRNA expression of angiogenic factors (e.g., Vegfa, Flt1, Klf4). Protein analysis identified an activation of anti-angiogenic mTOR effectors. In the postnatal phase, lung capillaries (< 20 µm) were significantly reduced, expression of CD31 and VE-Cadherin were unaffected, whereas SMAD1/5/8 signaling and Klf4 protein were increased (p < 0.01). Moreover, elevated proteolytic activity of MMP2 and MMP9 was linked to a 50% reduction of lung elastic fibres. In conclusion, we show a possible link of LBW in humans and reduced lung function in adulthood. Experimental IUGR identifies an *intrauterine phase* with inhibition of angiogenic signaling, and a *postnatal phase* with proteolytic activity and reduced elastic fibres.

## Introduction

Intrauterine growth restriction (IUGR) is a multifactorial disease affecting approximately 10% of the newborn population and is classically defined as a birthweight two standard deviations below the 50th percentile for the gestational age^1^. Recently, IUGR has been characterised as a failure of a foetus to reach its full growth potential due to genetic or environmental factors, including maternal, placental, and fetal causes, which then leads to restricted nutrient and oxygen supply^1,2^. Infants with IUGR are at higher risk for adverse perinatal complications, such as prematurity, respiratory distress, and bronchopulmonary dysplasia (BPD)^2^. It has also been demonstrated that IUGR followed by postnatal catch-up growth increases the susceptibility to chronic adult’s diseases beyond infancy, including hypertension and vasculopathies^3^.

Intrauterine exposure to adverse nutritional, metabolic, and hormonal alterations can interfere with organ development and induce life-long changes in structure and physiology. This was initially described as fetal, perinatal or metabolic programming by Barker and colleagues in the 1990s^4,5^. Previous studies have shown that IUGR impairs alveolar formation and lung growth, leading to reduced lung function in adult rats. These structural and functional changes may be related to a disruption of key developmental signaling pathways, such as TGFβ signaling, and inflammatory response. This may induce matrix remodeling, including distorted elastic fibre assembly^6,7^. While clinical studies associated IUGR with lower diffusion capacity, increased susceptibility to infections, and obstructive pulmonary disease^8–10^, the genetic disposition remain elusive. Recent studies demonstrated that low birth weight (LBW), used as a proxy for IUGR, is causally related to an increased susceptibility to coronary artery disease, and hightens the risk for pulmonary arterial hypertension (PAH) in infants with BPD^11^. These clinical data suggest a causual genetic association of IUGR and susceptibility to lung diseases that needs to be elucidated.

Angiogenesis is essential for alveolar formation and lung growth. The activation of endogenous pro-angiogenic pathways increases endothelial cell survival, proliferation and migration, thereby driving alveolarisation^12^. The concerted interaction of growth factor signaling, such as vascular endothelial growth factor (VEGF) and bone morphogenetic protein (BMP) signaling, is central in endothelial cell function^13,14^. The disruption of these critical angiogenic pathways impairs microvessel formation and arrests lung growth, as is seen in BPD^13,15^. Previous studies have shown that IUGR decreases pulmonary vessel growth in sheep and impairs proliferation, migration, and tube formation of pulmonary artery endothelial cells^16^. However, the molecular mechanisms disrupting developmental processes and angiogenesis in lungs following IUGR remain unclear.

**Figure 1:**
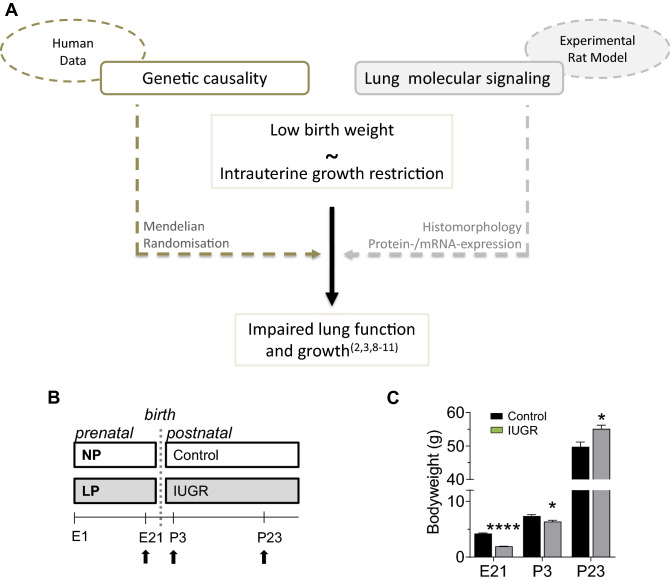
Schematic representation of the study layout, using Mendelian randomisation (left) as a strategy to study the genetic origin of low birth weight, vs. using molecular methods to study the effect of intrauterine growth restriction (IUGR) on lung development (right) (**A**). Timeline of the experimental model in which IUGR is induced with a low protein diet (LP) during gestation in Wistar rats; control dams received normal protein diet (NP). Lungs were harvested at embryonic day 21 (E21), postnatal day 3 (P3) and P23 (**B**). Measurements provide a quantification of the mean body weight of the control and IUGR group on time points E21, P3, and P23 (**C**). Mean ± SEM (n = 10/group). A non-parametric T-test was used to compare IUGR to the control group, *p < 0.05, ****p < 0.0001.

Based on the association of IUGR resulting in LBW and BPD we pursued two approaches: Firstly, we studied the long-lasting causal relationship of LBW and reduced lung function in humans with Mendelian randomisation; secondly, we investigated whether disruption of developmental pathways at intrauterine and postnatal time points contributes to impaired angiogenesis and elastic fibre metabolism after IUGR, thereby causing failed alveolarisation and functional impairment (Fig. 1A).

## Materials and methods

### Mendelian randomisation

We performed two-sample Mendelian randomisation analyses to infer causality between birth weight and forced expiratory volume in 1 s (FEV1) and forced vital capacity (FVC), used as proxies for lung function. We selected and used as exposure the genome-wide significant independent hits (p value ≤ 5E10−8) associated with own and offspring birth weight (Supplemental Table 1) from the genome-wide association meta-analyses of own and offspring birth weight performed by the Early Growth Genetics (EGG) Consortium^17^. We used the genome-wide association studies (GWAS) summary statistics of FEV1 (field ID 3063) and FVC (field ID 3062) performed in the UK Biobank by Neale et al. (http://www.nealelab.is/uk-biobank/) as outcomes.

We used four separate methods to estimate causal effects: the standard inverse-variance weighted (IVW) regression with and without MR-PRESSO^18^ (Mendelian Randomization Pleiotropy RESidual Sum and Outlier; to minimise the risk of horizontal pleiotropy); as well as two robust regression methods, the weighted median-based method, and Egger regression^19^. We applied robust methods with special assumptions about the behavior of pleiotropic variants, such as MR-Egger^20^, which assumes pleiotropic effects are uncorrelated with the genetic associations with the risk factor, the InSIDE assumption; and the MR-PRESSO^18^, that excludes outlying variants as being potentially pleiotropic. In addition, we performed leave-one-out sensitivity analyses to identify if a single SNP was driving an association. We estimated statistical power for the MR analyses assuming a clinically relevant fixed effect size of 0.1 SD with an alpha threshold of 0.05. Power for MR analyses was estimated using the method reported by Burgess et al.^21,22^. We performed the two-sample MR analyses^19,23^ with the R package *TwoSampleMR*.

### Animal studies

All animal procedures were performed as previously described^6,7^, in accordance with the German regulations and legal requirements and approved by the local government authorities (Regierung von Mittelfranken, AZ 621-2531.31-11/02 and AZ 621-2531.31-14/05)^24^. Three time points were investigated: (1) Embryonic day 21 (E21): caesarean section was performed and the foetuses were euthanised; (2) Postnatal day 3 (P3); (3) P23; 2–6 dams for each group. n = 10 for each experimental group. Lungs were excised *enblock* and either snap frozen and stored − 80 °C or fixed with 4% paraformaldehyde for paraffin embedding as previously described^7^.

### Tissue assays

#### RNA extraction and real-time qPCR

Total RNA extraction and quantitative RT-PCR were performed as previously described^7^. Quantitative changes in mRNA expression were assessed with Taqman or SYBR Green PCR Master Mix (Invitrogen, 11743500 & 11760500, Germany) using a 7500 Real-Time PCR System (Applied Bioscience). Primers were designed using Primer Express Software (Applied Biosystems) (Supplemental Table 2). The ΔΔ*C*_t_ method, as previously described, was used for quantification^25^.

#### Protein extraction, quantification, and immunoblot

Protein was isolated from homogenised whole-lung tissue and quantified, followed by immunoblotting as previously described^7^. The primary antibodies and the secondary peroxidase-conjugated anti-mouse, anti-rabbit or anti-rat antibodies (commercially available and tested) are listed in Supplemental Table 3. Quantitative analysis was performed with densitometry (Bio-Rad ImageLab software, Bio-Rad, Munich, Germany) using hypoxanthine–guanine phosphoribosyltransferase (HPRT) and β-Actin as loading controls. All data represent contiguous lanes, complete blots are shown in the Supplemental Figures.

#### Zymography

Protease activity of metalloproteinase 2 (MMP2) and MMP9 was analysed by gelatin-based zymography as previously described^7^. Activity was quantified by densitometric analysis on negative images (Image Lab Software, Bio-Rad Laboratories, Germany). All data represent contiguous lanes, blots are not cropped.

### Histology and immunhistochemistry

#### Microvessel count

Randomly selected lung sections (3 µm thickness) were stained with the primary antibody anti-von Willebrand Factor, followed by a secondary antibody (for details see Supplemental Table 3). The sections were then scanned using the slide scanner (Leica SCN400). Microvessels (2–20 µm and 20–100 µm, displaying a lumen) were quantified in a total of ten fields of 20× view per tissue section and 6 random tissue sections per animal (6 animals per group).

#### Elastic fibre quantification

Lung sections were stained for elastic fibres using Resorcin Fuchsin from Weigert (Weigert’s Iron Resorcin and Fuchsin Solution; Carl Roth, X877.3)^26^, and counterstained yellow with Tatrazine [0.5% in 0.25% acetic acid (Dianova, cat. no. TZQ999, USA)]. The elastic fibre density as an index of parenchymal elastin content was analysed in up to ten fields of 20× view per tissue section and 6 random tissue sections per animal (6 animals per group). Measurement was performed using Cell D 3.4 Olympus Soft Imaging Solutions (Olympus; CellSens, Germany).

### Statistical analysis

All results are displayed as mean ± SEM. The unpaired non-parametric T-test was used to compare the control (normal protein) to the IUGR (low protein) group. For a quantitative comparison between time points, a two-way ANOVA followed by Bonferroni post-test was performed. The p-value was adjusted using Bonferroni post-test and less than 0.05 was considered significant. The statistical analyses were carried out using the Graph Pad Prism software (GraphPad software Version 6.0 and 7.0, San Diego, CA, USA).

## Results

### MR analyses indicate a possible association of birth weight with adult lung function

With more than 80% statistical power our data suggest an association of birth weight with lung function (FVC and FEV1) in all Mendelian randomisation analyses performed. The leave-one out sensitivity analysis did not highlight any SNPs with a large effect on the results. After excluding outlying variants as being potentially pleiotropic using MR-PRESSO, the analysis showed neither significant heterogeneity or directional horizontal pleiotropy. The analyses using MR-Egger and weighted median methods consistently yielded similar effect estimates. The direction of the effect was positive (i.e., LBW was associated with reduced lung function). Full results are shown in Table 1 and Supplemental Figure 1.

**Table 1:** Mendelian randomisation analyses of birth weight with expiratory volume in 1 s (FEV1) and forced vital capacity (FVC).

Method	FVC				FEV1			
	nsnp	b	se	p	nsnp	b	se	p
**Own birthweight**								
IVW	220	0.214	0.024	**7.79E−20**	220	0.185	0.022	**4.56E−17**
MR-PRESSO	174	0.151	0.014	**6.02E−22**	180	0.132	0.014	**5.69E−18**
MR Egger	220	0.264	0.064	**5.59E−05**	220	0.218	0.060	**3.47E−04**
Weighted median	220	0.091	0.014	**1.07E−10**	220	0.059	0.014	**3.86E−05**
**Offspring birthweight**								
IVW	118	0.193	0.032	**2.25E−09**	118	0.183	0.030	**1.50E−09**
MR-PRESSO	82	0.121	0.017	**3.67E−10**	87	0.110	0.017	**2.95E−09**
MR Egger	118	0.276	0.102	**7.57E−03**	118	0.284	0.095	**3.42E−03**
Weighted median	118	0.067	0.017	**6.13E−05**	118	0.067	0.018	**2.40E−04**

The estimates represent SD change in outcome variable per SD change in the exposure tested. P-values significant after Bonferroni correction (p ≤ 1.25*10^−1^) are bold.

se, standard error; P, p-value; nsnp, number of single-nucleotide polymorphism; IVW, inverse variance weighted method.

### IUGR was established in a low-protein diet rat model

Low protein diet of dams during gestation induced IUGR in the offspring when compared to controls. The experimental rat model of IUGR is illustrated in Fig. 1B. As shown in Fig. 1C, offspring had LBW at (E21) and P3, whereas their weight was significantly higher at P23, indicating a postnatal catch-up growth after IUGR.

### IUGR alters pulmonary vessel growth and regulates endothelial cell markers

**Figure 2:**
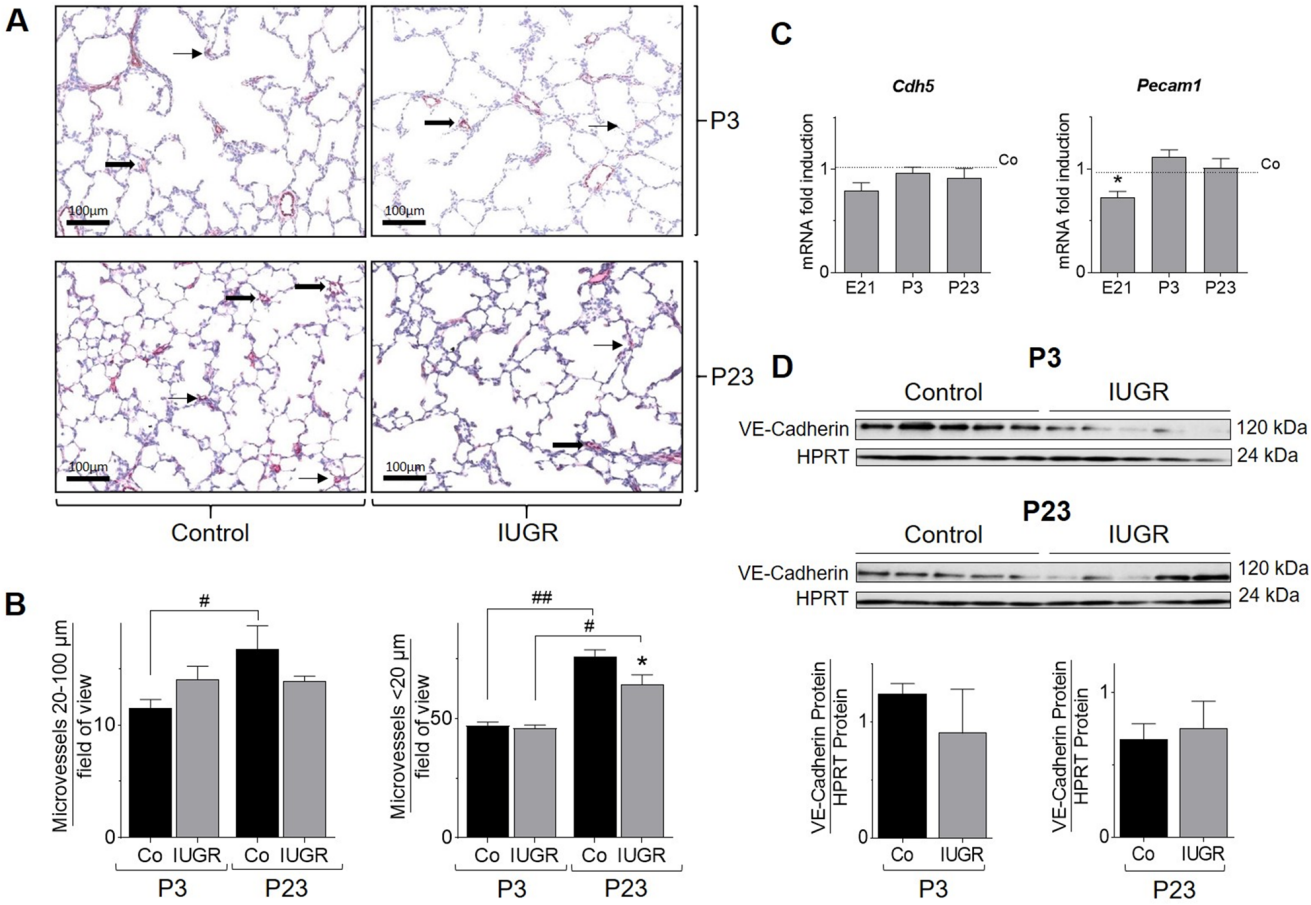
Intrauterine growth restriction (IUGR) impairs angiogenesis. (**A**,** B**) Representative images (×20  magnification) of pulmonary microvessels (0–100 µm) on postnatal day 3 (P3), and on postnatal day 23 (P23). Random lung sections were stained with von Willebrand Factor as an indicator of endothelial cells, followed by counting of microvessel count for vessels with a diameter of 20-100 µm (marked with  ), and vessels with a diameter < 20 µm (marked with  ). The respective quantification is shown below the immunhistochemical stainings (n = 6/group). (**C**) Assessment of mRNA expression of pulmonary endothelial cell markers, VE-Cadherin (*Cdh5*) and CD31 (*Pecam1*) on embryonic day 21 (E21), P3 and P23 using qRT-PCR; Glyceraldehyde 3-phosphate dehydrogenase (*Gapdh*) served as housekeeping gene; the control group was set at 1. (**D**) Measurement of protein abundance using immunoblot; the respective densitometric quantification of protein expression of VE-Cadherin on P3 and P23 is shown below the immunblots; VE-Cadherin was related to the loading control HPRT (n = 5–6/group). Mean ± SEM. A non-parametric T-test was used to compare IUGR to the control group,*p < 0.05. Comparison between P3 and P23 with a two-way ANOVA test, ^#^p < 0.05, ^##^p < 0.01.

Quantification of lung microvessels with CD31 immunostaining at P3 and P23 (Fig. 2A) revealed a significant formation of larger microvessels (20–100 µm) between P3 und P23 in control pups, not visible in IUGR. Small microvessels (< 20 µm) were significantly decreased in IUGR when compared to control at P23, suggesting dysfunctional postnatal angiogenesis after IUGR (Fig. 2B). The mRNA expression of *Pecam1* (CD31) was significantly reduced at E21 and immunoblot showed a non-significant reduction of VE-Cadherin protein expression at P3, but not at P23 after IUGR, compared to control (Fig. 2C,D).

### IUGR dynamically regulates the pro-angiogenic VEGF pathway in lungs

**Figure 3:**
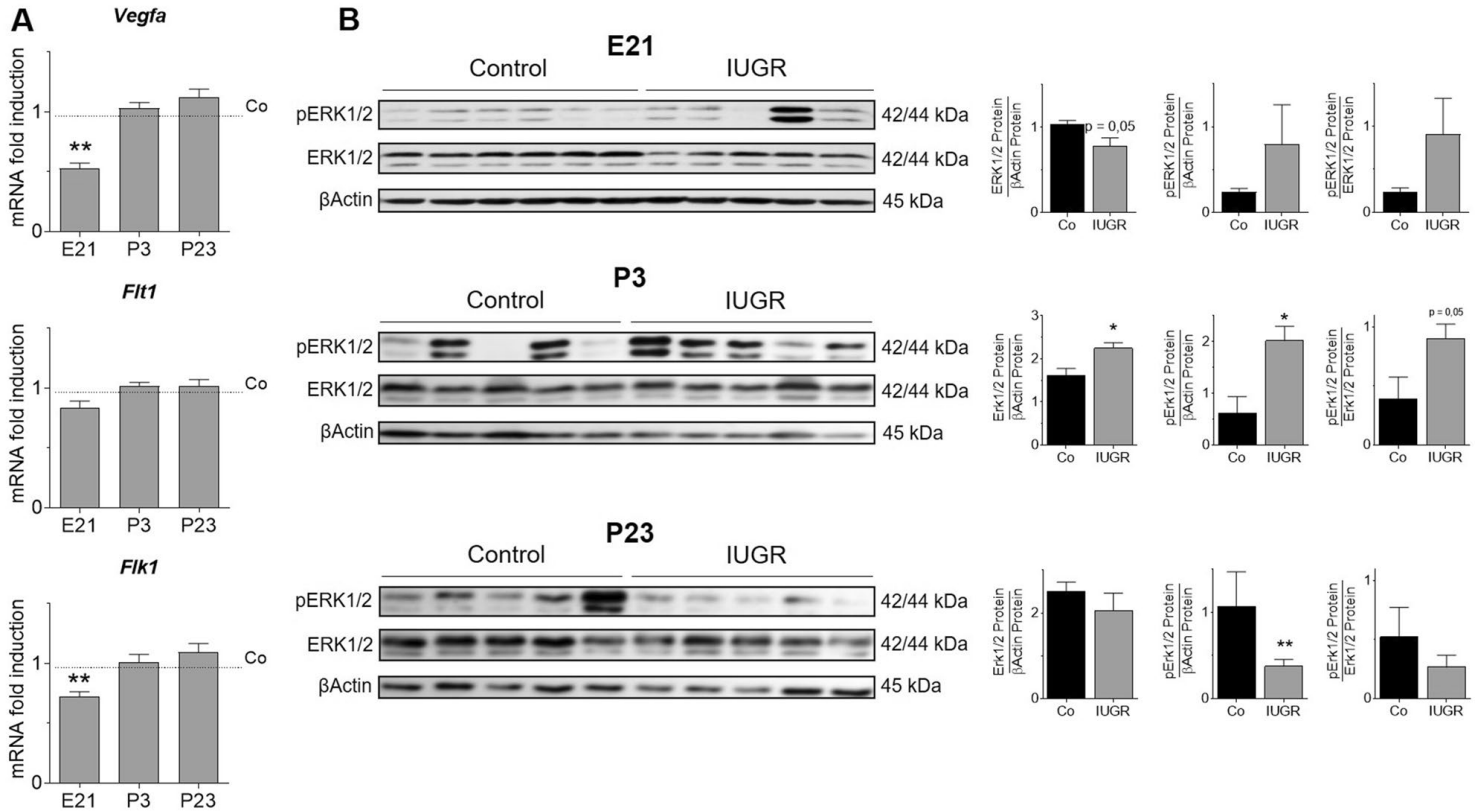
IUGR dysregulates gene expression of components and the activation of vascular endothelial growth factor (VEGF) signaling in lungs. (**A**) Assessement of *Vegfa*, VEGF-Receptor 1 (*Flt1*) and VEGF-R2 (*Flk1*) on embryonic day (E21), postnatal day 3 (P3), and P23 using qRT-PCR; Glyceraldehyde 3-phosphate dehydrogenase (*Gapdh*) served as housekeeping gene; the Control group was set at 1 (n = 10/group). (**B**) Immunoblots illustrating protein abundance of total ERK1/2 and phosphorylated ERK1/2 (pERK1/2) on E21, P3 and P21; pERK1/2 was related to βActin or to total ERK1/2; the densitometric analysis is shown next to the respective immunoblot (n = 5–6/group). Mean ± SEM. A non-parametric T-test was used to compare IUGR to the control group,*p < 0.05, **p < 0.01.

The mRNA expression of the ligand *Vegfa* as well as its receptors, VEGF-R1 (*Flt1*) and VEGFR2 (*Flk1*), were significantly reduced in lungs after IUGR at E21, but not at P3 and P23 (Fig. 3A). The analysis of the VEGF-signaling pathway ERK1/2 revealed a significant reduction of total ERK1/2 at E21, with slightly increased phosphorylated ERK1/2 (pERK1/2) relative to total ERK1/2. Early postnatal (P3), IUGR increased total ERK1/2 protein as well as pERK1/2. Finally, we detected an inhibition of pERK1/2 in lungs after IUGR when compared to controls at the late postnatal stage (P23) (Fig. 3B).

### IUGR dysregulates the pro-angiogenic BMP signaling pathway in lungs

**Figure 4:**
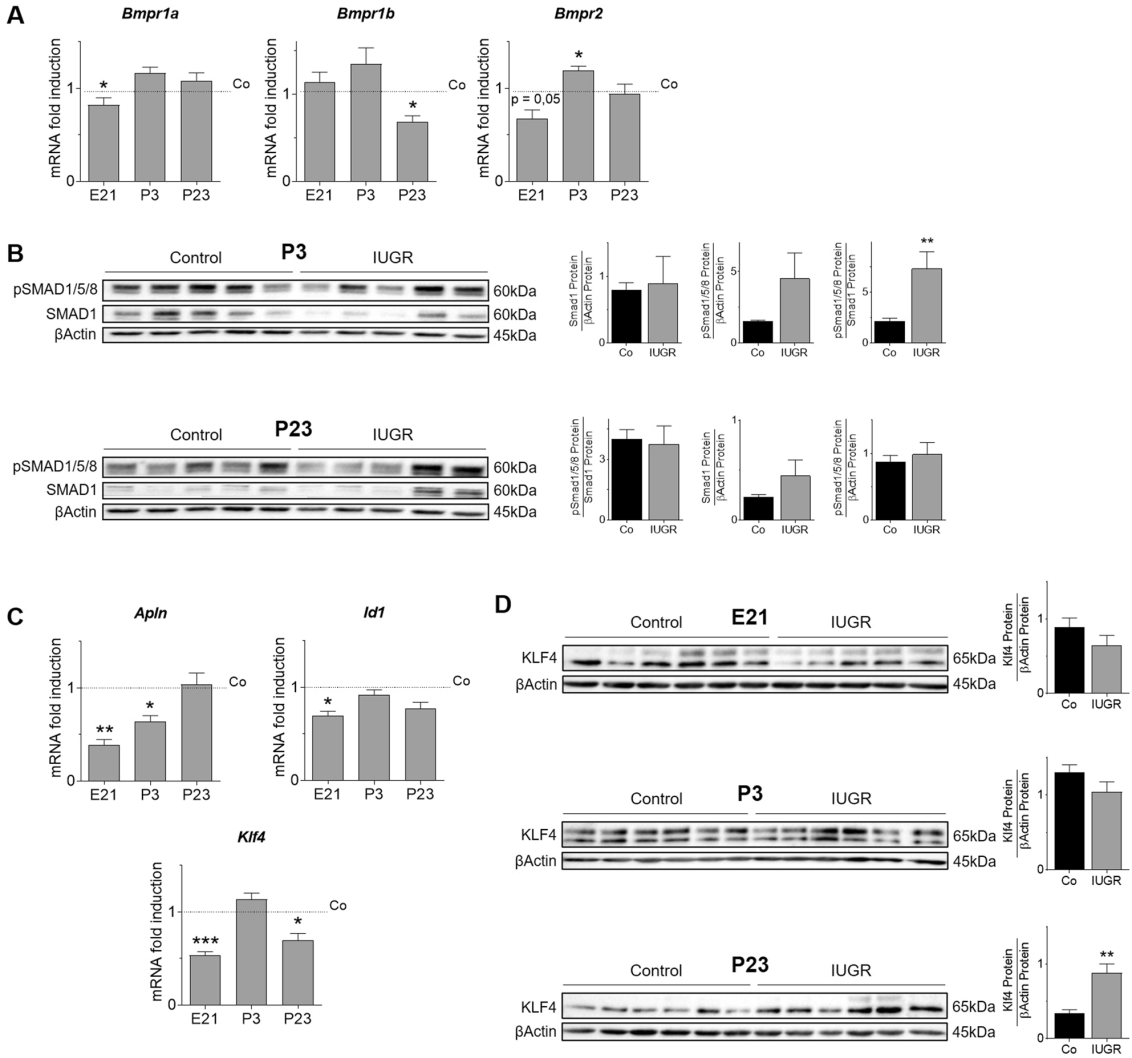
IUGR dysregulates gene expression and activation of the bone morphogenetic protein (BMP)-signaling pathway components. (**A**) Assessement of gene expression of BMP-Receptor 1a *(Bmpr1a), Bmpr1b* and *Bmpr2* on embryonic day (E21), postnatal day 3 (P3), and P23 using qRT-PCR; Glyceraldehyde 3-phosphate dehydrogenase (*Gapdh*) served as housekeeping gene; the Control group was set at 1 (n = 10/group). (**B**) Immunoblots showing protein abundance of phosphorylated SMAD1/5/8 (pSMAD1/5/8) and total SMAD1 on P3 and P23; βActin served as loading control; pSMAD1/5/8 was related to βActin or to total SMAD1; densitometric summary data are shown next to the respective immunoblot (n = 5–6/group). (C) Measurement of mRNA expression of angiogenic transcription factors apelin (*Apln*), inhibitor of differentiation 1 (*Id1*), and Krüppel-like factor 4 (*Klf4*); *Gapdh* served as housekeeping gene; the Control group was set at 1 (n = 10/group) (**C**), as well as the protein expression of transcription factor Klf4 (**D**), with densitometric quantification shown below (n = 5–6/group). Mean ± SEM. A non-parametric T-test was used to compare IUGR to the control group, *p < 0.05, **p < 0.01, ***p < 0.001.

BMP-receptor1a (*Bmpr1a*) was significantly reduced at E21, whereas *Bmpr1b* mRNA expression was significantly reduced after IUGR at P23. *Bmpr2* mRNA was also expressed differentially, with a slight reduction at E21 and a significant upregulation at P3 after IUGR (Fig. 4A). Immunoblot of the downstream signaling cascade of BMPRs showed activation of SMAD1/5/8 (pSMAD1/5/8) after IUGR at P3, but no difference at P23 (Fig. 4B). The mRNA expression of apelin (*Apln*), inhibitor of differentiation (*Id1*), and Krüppel-like factor 4 (*Klf4*), regulators of endothelial cell homeostasis or BMP interactors, were also reduced after IUGR at E21. *Apln* remained significantly lower in IUGR than Control at P3, whereas *Klf4* gene expression was significantly downregulated at P23 (Fig. 4C). The protein abundance of Klf4, a regulator of stem cell capacity, was slightly reduced at E21 and more than twofold increased at P23 (Fig. 4D).

### IUGR dysregulates cell metabolism through AMPKα and mTOR signaling

**Figure 5:**
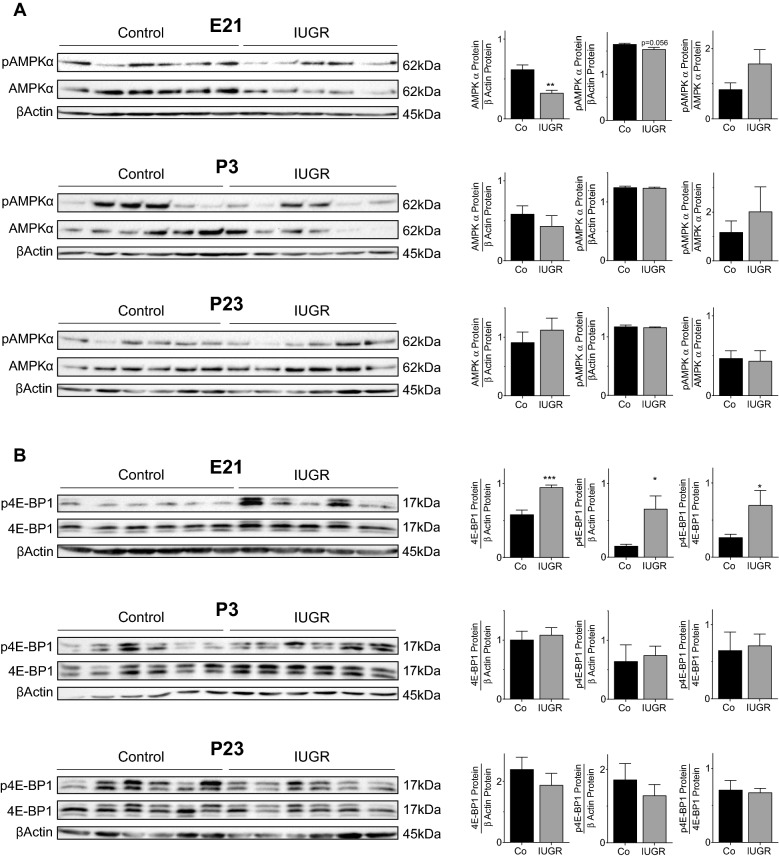
IUGR dysregulates nutrient-sensing signaling pathways that are involved in angiogenesis. (**A**) Immunoblots showing the analysis of AMP-Activated protein kinase (AMPK)α-pathway in total lung homogenate on embryonic day (E21), postnatal day 3 (P3), and P23; total AMPKα and phosphorylated AMPKα (pAMPKα) were assessed; βActin served as loading control; pAMPKα was related to β-Actin or to total AMPKα; densitometric summary data are shown next to the respective immunoblot (n = 6/group). (**B**) Total 4E-BP1 and phosphorylated 4E-BP1 (p4E-BP1) as a downstream effector of mTOR-pathway were assessed with immunoblot on E21, P3, and P23; βActin served as loading control; p4E-BP1 was related to βActin or to total 4E-BP1; densitometric summary data are shown next to the respective immunoblot (n = 6/group). Mean ± SEM. A non-parametric T-test was used to compare IUGR to the Control group, *p < 0.05, **p < 0.01, ***p < 0.001.

Immunoblot analysis revealed that IUGR significantly inhibited AMPKα signaling during the intrauterine phase (E21) (Fig. 5A). In contrast, assessment of mTOR signaling using 4E-BP1 as a downstream effector showed a marked activation at E21 (Fig. 5B). However, during postnatal lung development neither AMPKα nor mTOR signaling were significantly regulated by IUGR.

### IUGR increases lung protease activity and reduces lung elastic fibre content

**Figure 6:**
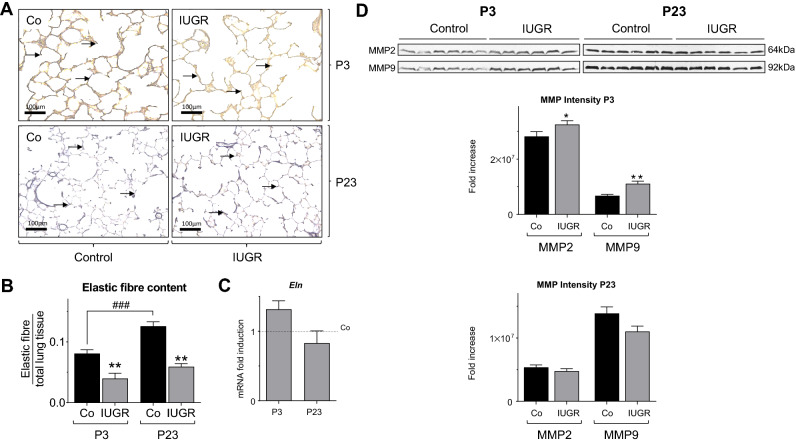
IUGR activates lung proteolytic activity and reduces lung elastic fibre content. (**A**) Representative images (20x  magnification) of Hart stained lung at postnatal day 3 (P3) and P23, depicting elastic fibres as indicated with black arrows. (**B**) The quantification of elastic fibre content, relative to total lung tissue at P3 and P23 (n = 6/group). (**C**) Measurement of Elastin (*Eln*) mRNA expression on P3 and P23 using qRT-PCR analysis; Glyceraldehyde 3-phosphate dehydrogenase (*Gapdh*) served as housekeeping gene; the Control group was set at 1 (n = 10/group). (**D**) Analysis of the proteolytic activity of matrix metalloprotease 2 and 9 (MMP2, MMP9) at P3 and P23 using zymography. Both MMP2 and MMP9 are regulators of matrix remodelling; the densitometric quantification of the zymography is shown below (n = 6/group). Mean ± SEM. A non-parametric T-test was used to compare IUGR to the control group, *p<0.05, **p < 0.01. Comparison between P3 and P23 with a two-way ANOVA test, ^###^p < 0.001.

A Hart stain for elastic fibres (Fig. 6A) revealed a reduction of relative elastic fibre content by 50% at both time points P3 and P23 (Fig. 6B). Assessment of *Eln* (elastin) mRNA expression did not significantly differ between IUGR and Control group (Fig. 6C), suggesting increased degradation as a result of proteolytic activity. To test this notion we used zymography, and determined a significant increase of metalloproteinase 2 (MMP2) and MMP9 activity after IUGR at P3 (Fig. 6D).

## Discussion

The present study shows a possible association between LBW in humans, used as a proxy of IUGR, and reduced lung function in adulthood. In addition, it provides a pathomechanism of acute and long-term impact of IUGR on pulmonary microvascular formation and elastic fibre formation in an experimental rat model of IUGR. The combined results of these two approaches paint the picture of dynamic intrauterine programming of pulmonary development. Specifically, during the *intrauterine phase,* restricted nutrient supply and cellular stress are related to a significant disruption of pro-angiogenic VEGFA- and BMP-signaling, activation of anti-angiogenic mTOR signaling, downregulation of angiogenic growth and transcription factors, and loss of endothelial cell markers. Whereas during the *postnatal phase*, angiogenic signaling caught up, but small microvessels were significantly reduced and associated with an increased lung proteolytic activity, resulting in a persistent reduction of elastic fibre content. These processes accumulate in a loss of microvasculature and alveolarisation, evidenced by a loss of lung function in adulthood.

### Linking LBW to reduced lung function using Mendelian randomization

With Mendelian randomisation, we have shown a positive association between LBW and reduced lung function (FEV1, FVC), indicating a possible reduced functional lung volume in individuals with LBW. It has been previously shown that a reduction of FVC is associated with lung emphysema^27^, defined as a decrease in elastic recoil^28^. In addition, previous studies have shown an association between LBW and type 2 diabetes and cardiovascular diseases^11^. Our data provide a direct link between maternal effects and LBW-associated lung function that is in line with the Barker Hypothesis.

### Strengths and limitations of Mendelian randomization

Our study included the large sample size and the most recent and powerful GWAS summary statistics used as exposures and outcomes. In addition, we used a wide range of sensitivity analyses increasing robustness of our findings. Our study also has several limitations. First, the participants included in the genetic analyses were of European ancestry. Hence, our results may not be generalizable to other ethnic groups with significantly different prevalence/predispositions with regard to the outcome. Second, we analyzed birth weight as a continuous variable without specifically looking at LBW. Moreover, we cannot exclude the importance of additional environmental factors implicated in the relationship between birth weight and lung function. Finally, samples of the GWAS for the exposure (the genome-wide association meta-analyses of own and offspring birth weight) and the GWAS for the outcome [FEV1 (field ID 3063) and FVC (field ID 3062) performed in the UK Biobank] have in part some overlaps and could thereby potentially bias our results.

### Translational approach: dysregulation of angiogenic signaling is associated with impaired lung growth in rats with IUGR

VEGF promotes angiogenesis and alveolarisation in animal models of neonatal lung injury^29^. In the present study, we demonstrate a significant downregulation of *Vegfa* and *Flk1* (VEGF-R2) in the intrauterine phase, which is followed by a catch-up to reach Control levels. Interestingly, the downstream effector ERK1/2 protein^30^ is reduced on E21, and increased after birth. However, the activation of ERK1/2 remains reduced at P23, suggesting a possible programming of intracellular VEGF-A resistance. Moreover, VEGF signaling is also activated by AMPK, a pathway that promotes endothelial cell differentiation, proliferation and migration^31^. Reduced activation of the AMPKα signaling pathway during the intrauterine period may further contribute to the inhibition of the VEGF signaling machinery.

BMP signaling drives angiogenesis and is essential for the maintenance of endothelial cell homeostasis, and mutations in this family are associated with hereditary PAH. The transduction of BMP signaling is facilitated by complex formation between BMP receptors 1 and 2, activating its downstream canonical SMAD1/5/8 and non-canonical ERK1/2 signaling^14^. Our data show that IUGR significantly downregulates *Bmpr1a* and *Bmpr2* during the intrauterine period, whereas *Bmpr1b* was unaffected. In the postnatal phase, we found opposing effects, with similar gene expression of *Bmpr1a* and *Bmpr2*, but significant lower *Bmpr1b* in IUGR than control lungs. IUGR may block the proliferative Bmpr1a/Bmpr2 pathways during the intrauterine phase, while it is shifted to pro-proliferative signaling by postnatal reduction of *Bmpr1b* as an attempt to promote vascularisation and compensate for the intrauterine growth restriction^32^. This notion is further sustained by a postnatal activation of the downstream effector pSMAD1/5/8. This early developmental blockade of VEGF and BMP-receptor signaling in the lung may impair angiogenesis, induce arrest of alveolarisation and contribute to early origin of chronic lung diseases, such as BPD and PAH.

To determine whether the disruption of developmental signaling after IUGR may be related to changes in growth and transcription factors, we assessed *Id1*, *Apln*, and *Klf4* expression. In accordance with our previous data, *Id1*, *Apln*, and *Klf4* were significantly reduced by 50% in fetal lungs with IUGR, and postnatally upregulated. Downregulation of these factors has been associated with the pathogenesis of PAH^15,33,34^. The growth factor apelin is pro-angiogenic and regulates endothelial cell migration, proliferation and survival^15^, and preserves lung growth in a model of BPD^35^. Similarly, the expression of *Id1* is induced by BMPR2 signaling, and maintains endothelial cell function^33,36^. On the other hand Klf4, promotes angiogenesis through activation of VEGF signaling^34^, and regulates cell survival and differentiation. Loss of these angiogenic factors may impair vascular development and induce chronic vascular lung disease.

### Linking nutrient sensing pathways to angiogenic signalling in lungs of rats with IUGR

The pathways that signal anti-angiogenic effects during lung development remain uncertain. Organ growth requires an increased metabolic rate, which requires oxygen and nutrient supply. Nutritive restriction or placental insufficiency forces adaptation of the metabolism by changing cellular energy consumption. The serine/therosine kinase, mammalian target of rapamycin (mTOR) senses nutrient status in growing vessels, and modulates cellular responses during angiogenesis^37^. For example, rapamycin inhibits VEGF synthesis, has antiproliferative activity, and blocks angiogenesis in vivo^37^. Likewise, it was shown that mTOR signaling regulates angiogenic sprouting^38^. Our data supports the notion that activated mTOR signaling in the intrauterine phase may interact with BMP, and disrupt angiogenic signaling after IUGR.

### Proteolytic activity and disruption of elastic fiber formation may contribute to impaired lung structure and function after IUGR

Despite the postnatal catch-up of angiogenic signaling after IUGR, the number of small microvessels remains reduced. This initial paradox let us study processes independent of angionic signaling. Lung matrix is crucial in alveolarisation and serves as a scaffold that directs secondary septation and microvascular formation^39^. Experimental studies demonstrate that microvascular formation is reduced in genetically modified mice with elastin haplo-insufficiency, possibly increasing the risk for PAH^40^. Similarly, disturbed elastic fibre assembly and distribution are related to inhibition of angiogenesis in animal models of BPD^41^. BMPR2 signaling might contribute to impaired elastic fibre assembly via the BMP4-TGFβ1 pathway^40,42^. Additionally, we show a postnatal activation of metalloproteinases MMP2 an MMP2, linked to 50% reduction of elastic fibre content, persisting until P23. Increased degradation of elastic fibres might contribute to the post-IUGR alteration of lung function throughout life, including reduced FVC and increased FEV1. While our model of nutrient deprivation is highly relevant in countries, in which pregnant women are exposed to malnutrition, it does not reflect the pathomechanisms of placental insufficiency. In future studies, we recommend to confirm our findings in other models of IUGR. With our translational data from genome wide association studies, however, we aimed to correct for all causes of IUGR by defining a genetic association between LBW and lung function, independent of the cause, in a cross-sectional cohort.

## Conclusion

**Figure 7:**
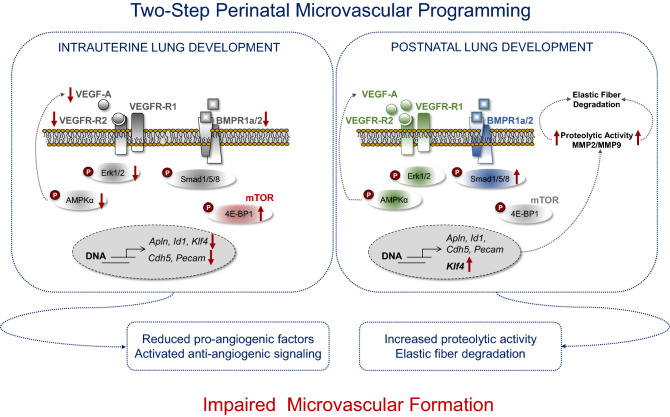
Speculative working model of two-step perinatal microvascular programming after intrauterine growth restriction (IUGR) with low birth weight (LBW). Fetal genetic factors contribute to low birth, linked to reduced lung function later in life. *Intrauterine phase*: IUGR inhibits angiogenic signaling, including vascular endothelial growth factor (VEGF), AMP-Activated protein kinase (AMPK)α, and bone morphogenetic protein (BMP) signaling, whereas the anti-angiogenic mechanistic Target of Rapamycin (mTOR) signaling is activated. *Postnatal phase*: transient activation of angiogenic signaling; the loss of elastic fibres is associated with increased proteolytic activity after IUGR. Both intrauterine and postnatal phase are related to reduced microvascular formation in lungs after IUGR and could account for the reduced lung function determined in infants born with low birth weight using Mendelian randomisation.

In conclusion, our translational data show a possible association between low birth weight and lung function. Our study provides novel insight as to how IUGR disrupts lung angiogenic signaling, elastic fibre formation, and microvascular formation in a time dependent manner in an experimental rat model. Using this approach, we identified two phases of perinatal vascular programming (Fig. 7): *first*, an intrauterine phase with inhibition of angiogenic signaling; and *second*, a postnatal phase, in which vascular signaling catches up, but lack of formation of small microvessels is related to increased proteolytic activity, and ultimately marked reduction of elastic fibre content. These data highlight how adverse intrauterine malnutrition programs the microvascular system, increasing the susceptibility to chronic lung diseases such as BPD and PAH.

## Supplementary Information

Supplementary Information.
