# Supplementary material for: Mendelian randomization and experimental IUGR reveal the adverse effect of low birth weight on lung structure and function
Source: Sci Rep. 2020 Dec 28;10:22395. doi: 10.1038/s41598-020-79245-7 (PMC7769986; doi:10.1038/s41598-020-79245-7)
Supplement: Supplementary file 1 — Supplementary Information. [file 41598_2020_79245_MOESM1_ESM.docx]

**Mendelian randomization and experimental IUGR reveal**

**the adverse effect of low birth weight on lung structure and function**

Celien Kuiper-Makris^1,2^, Daniela Zanetti^3,4^, Christina Vohlen^1,2^, Luise Fahle^1,2^, Marion Müller^5^, Margarete Odenthal^5,6^, Ursula Felderhoff-Müser^7^, Jörg Dötsch^2^, Miguel A. Alejandre Alcazar^1,6,8,9^.

^1^University of Cologne, Faculty of Medicine and University Hospital Cologne, Translational Experimental Pediatrics - Experimental Pulmonology, Department of Pediatric and Adolescent Medicine, Germany; ^2^University of Cologne, Faculty of Medicine and University Hospital Cologne, Department of Pediatric and Adolescent Medicine, Germany; ^3^Department of Medicine, Division of Cardiovascular Medicine, Stanford University School of Medicine, Stanford, CA; ^4^Stanford Cardiovascular Institute, Stanford University, Stanford, CA; ^5^University of Cologne, Faculty of Medicine and University Hospital Cologne, Institute of Pathology, Germany; ^6^University of Cologne, Faculty of Medicine and University Hospital Cologne, Center for Molecular Medicine Cologne (CMMC), Germany; ^7^Dept. of Paediatrics I, University Hospital Essen, University Duisburg-Essen; ^8^Institute for Lung Health, University of Giessen and Marburg Lung Centre (UGMLC), Member of the German Centre for Lung Research (DZL), Gießen, Germany; ^9^Cologne Excellence Cluster for Stress Responses in Ageing-associated diseases (CECAD).

**Corresponding author:** Miguel A. Alejandre Alcázar, M.D., Ph.D.

Assistant Professor (Translational Experimental Pediatrics)

Experimental Pulmonology, Department of Pediatrics and Adolescent Medicine, University of Cologne

Kerpener Strasse 62

D-50937 Cologne

Tel.: +49 (0)221 478 96876

Fax.: +49 (0)221 478 96868

Email: miguel.alejandre-alcazar@uk-koeln.de

All authors declare that there is no conflict of interest concerning this study.

**Supplemental Figure legends**


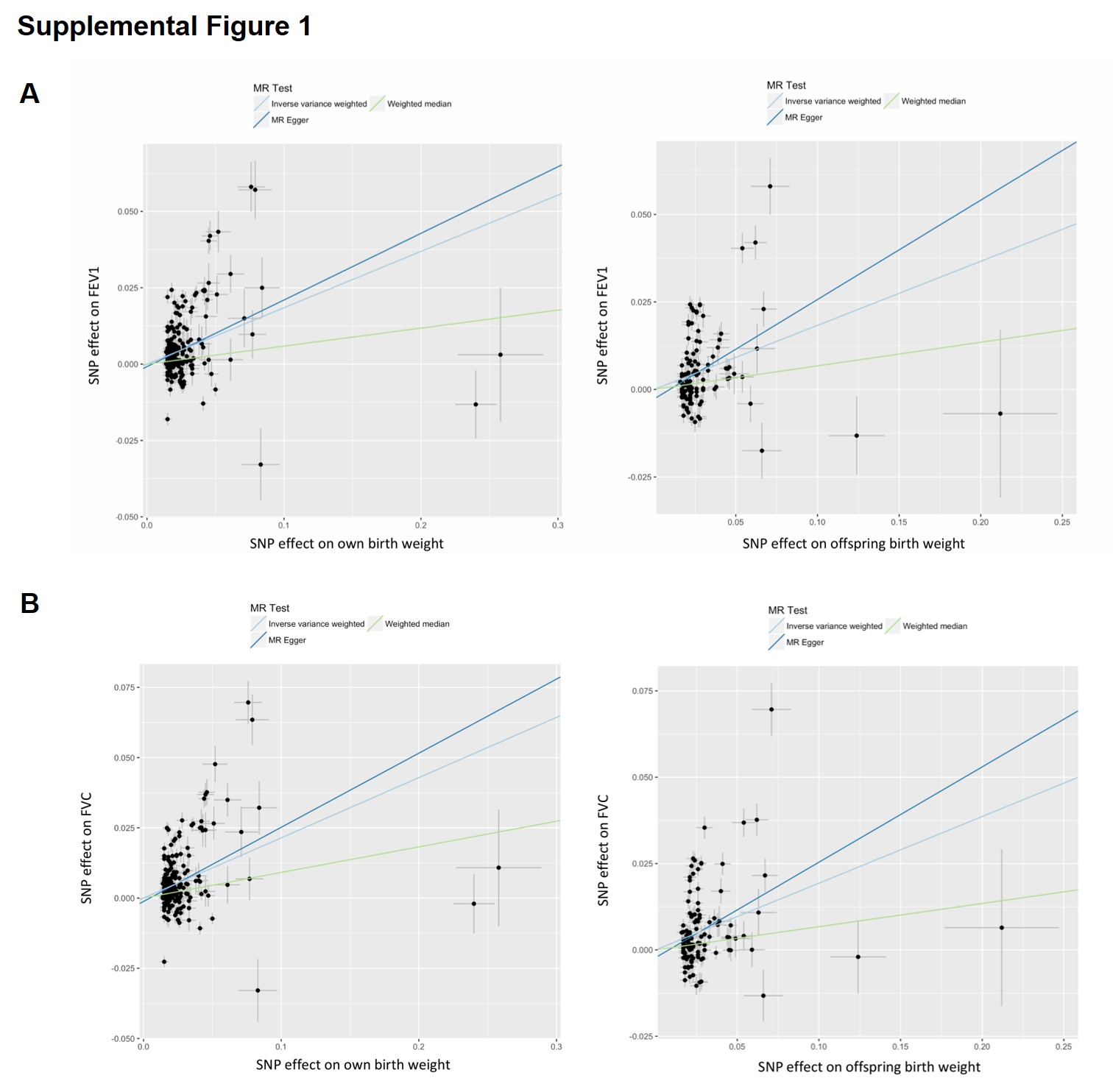


**Supplemental Figure 1.** Scatter plots of the effect of genetic genetic variants on own birth weight and offspring birth weight using forced expiratory volume in 1 second (FEV1) (A) and forced vital capacity (FVC) (B) as proxies. The causality between the effect of birth weight on lung function was tested with three different methods: inverse-variance weighted regression, weighted median-based method and Egger regression.


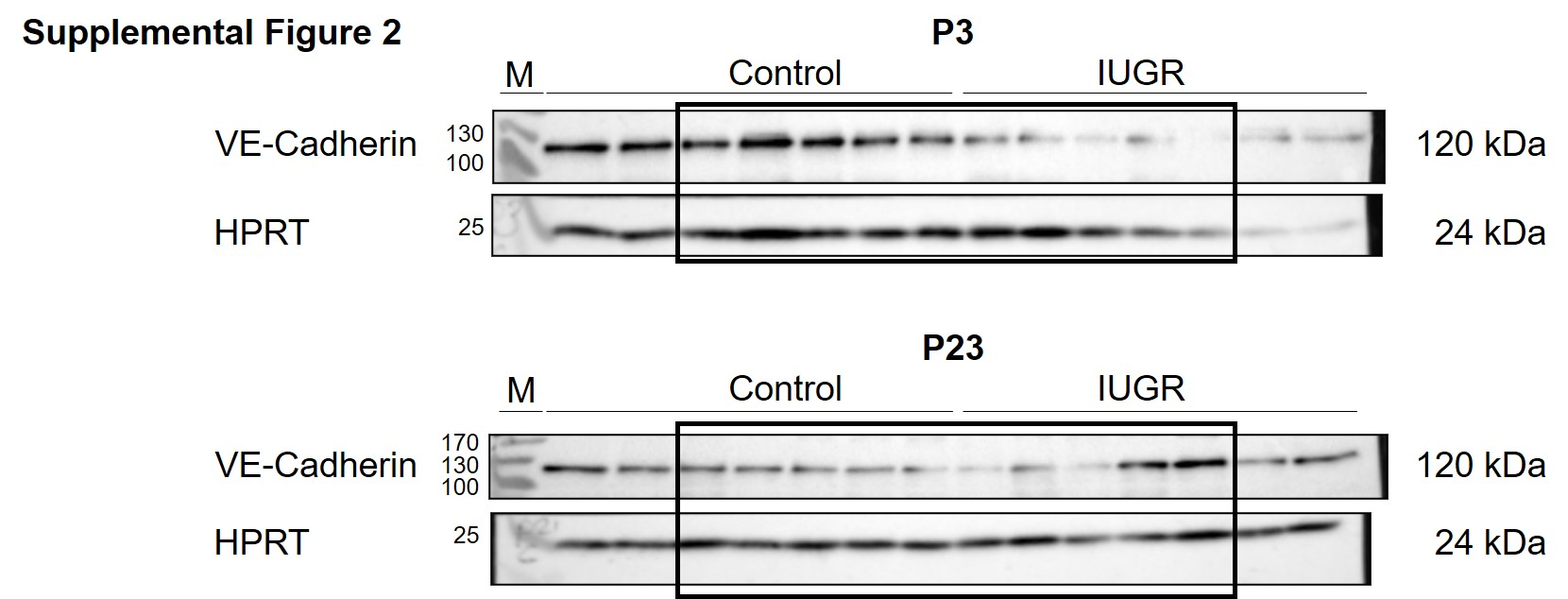


**Supplemental Figure 2.** Complete picture of immunoblots, as partly depicted in Figure 2D. Markers for molecular weight (M) are depicted on the left and right end of the blot, the molecular weight is represented in kDa. The protein expression of VE-Cadherin on P3 and P23 are shown; VE-Cadherin was related to the loading control β-Actin (n=7/group).


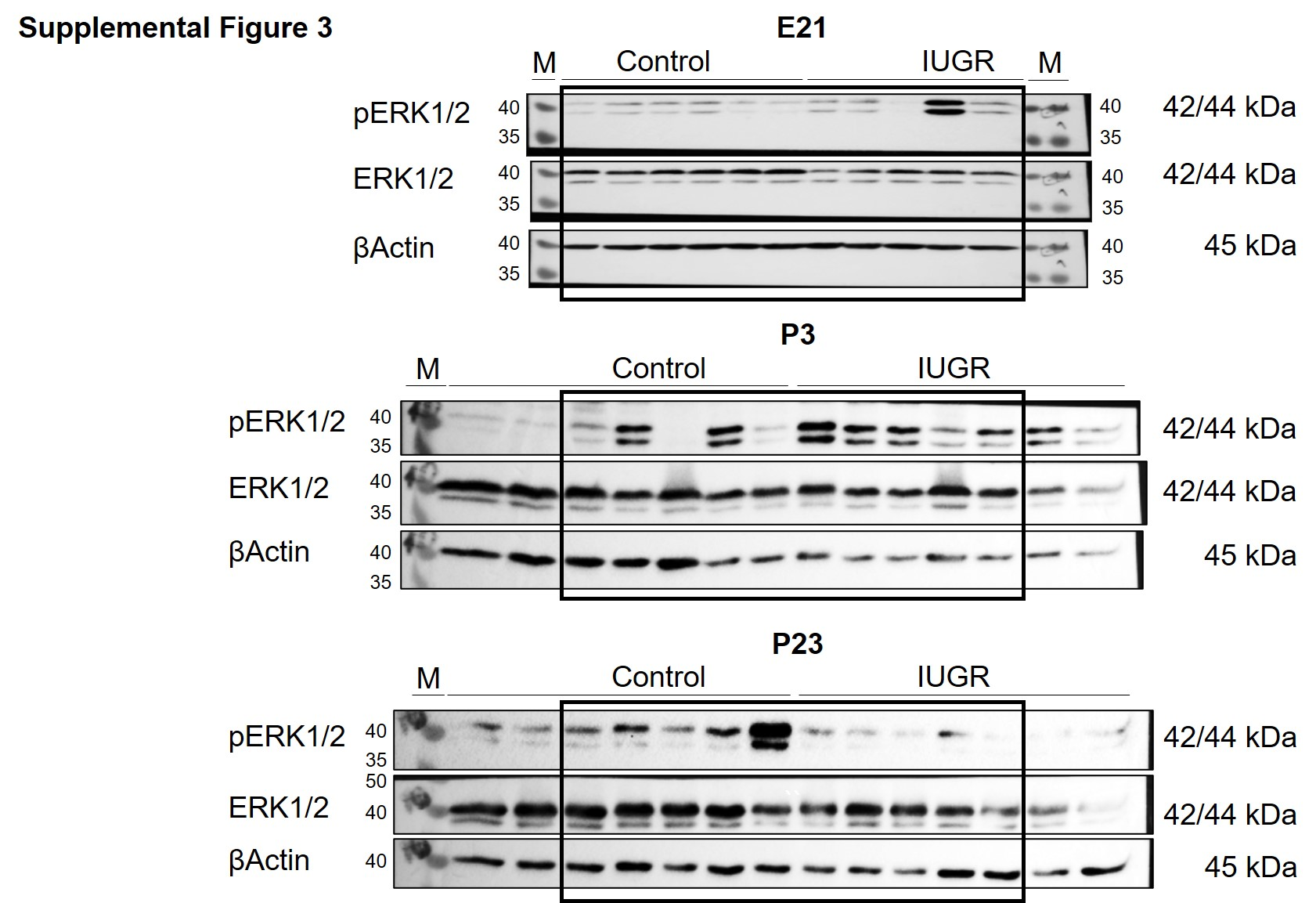


**Supplemental Figure 3.** Complete picture of immunoblots, as partly depicted in Figure 3B. Markers for molecular weight (M) are depicted on the left (and at E21 on the right) end of the blot, the molecular weight is represented in kDa. Immunoblots illustrate the protein abundance of total ERK1/2 and phosphorylated ERK1/2 (pERK1/2) on E21, P3 and P21 (n=5-7/group).


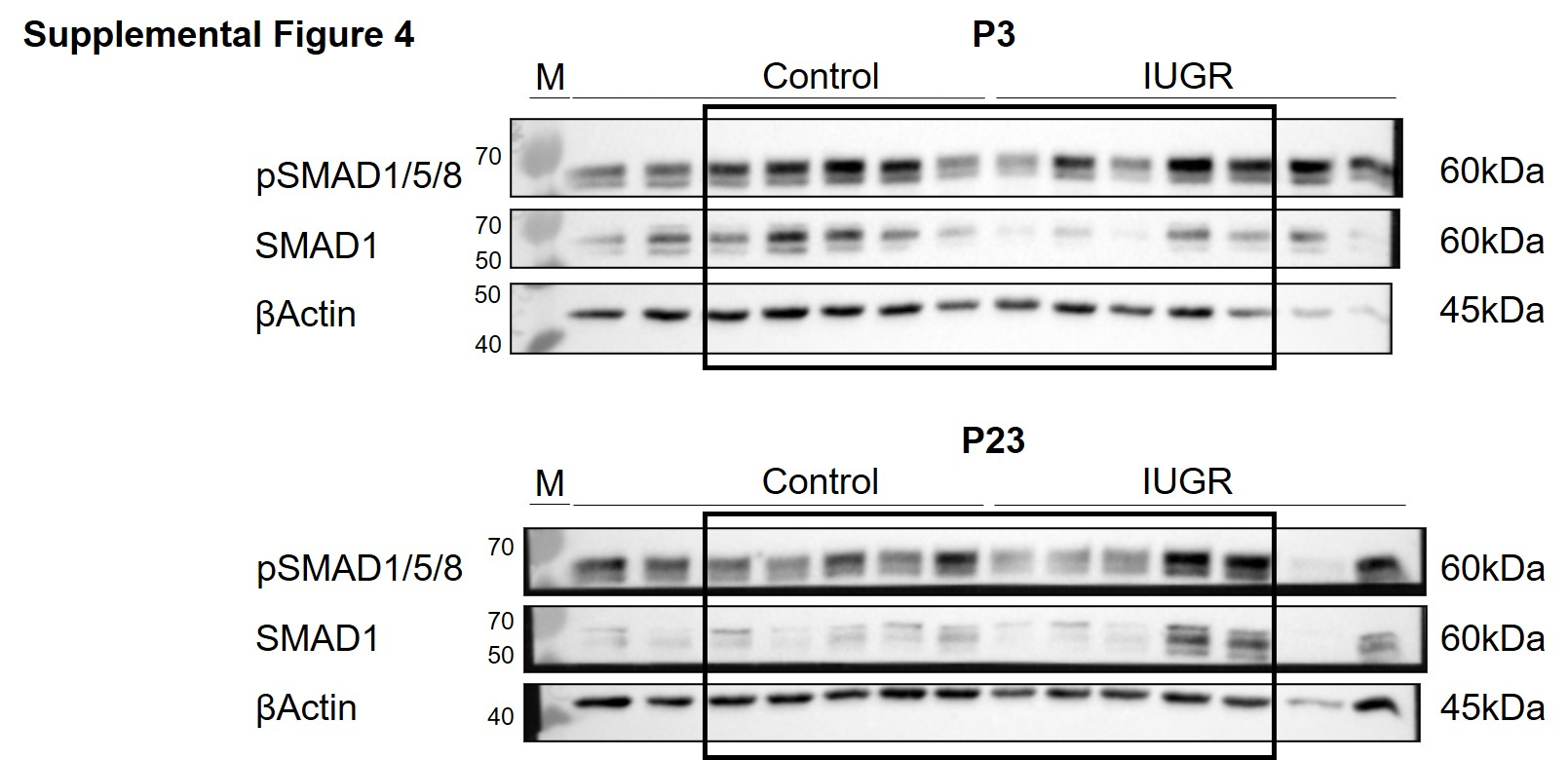


**Supplemental Figure 4.** Complete picture of immunoblots, as partly depicted in Figure 4B. Markers for molecular weight (M) are depicted on the left end of the blot, the molecular weight is represented in kDa. The protein abundance of phosphorylated SMAD1/5/8 (pSMAD1/5/8) and total SMAD1 on P3 and P23 is shown; β-Actin served as loading control (n=7/group).


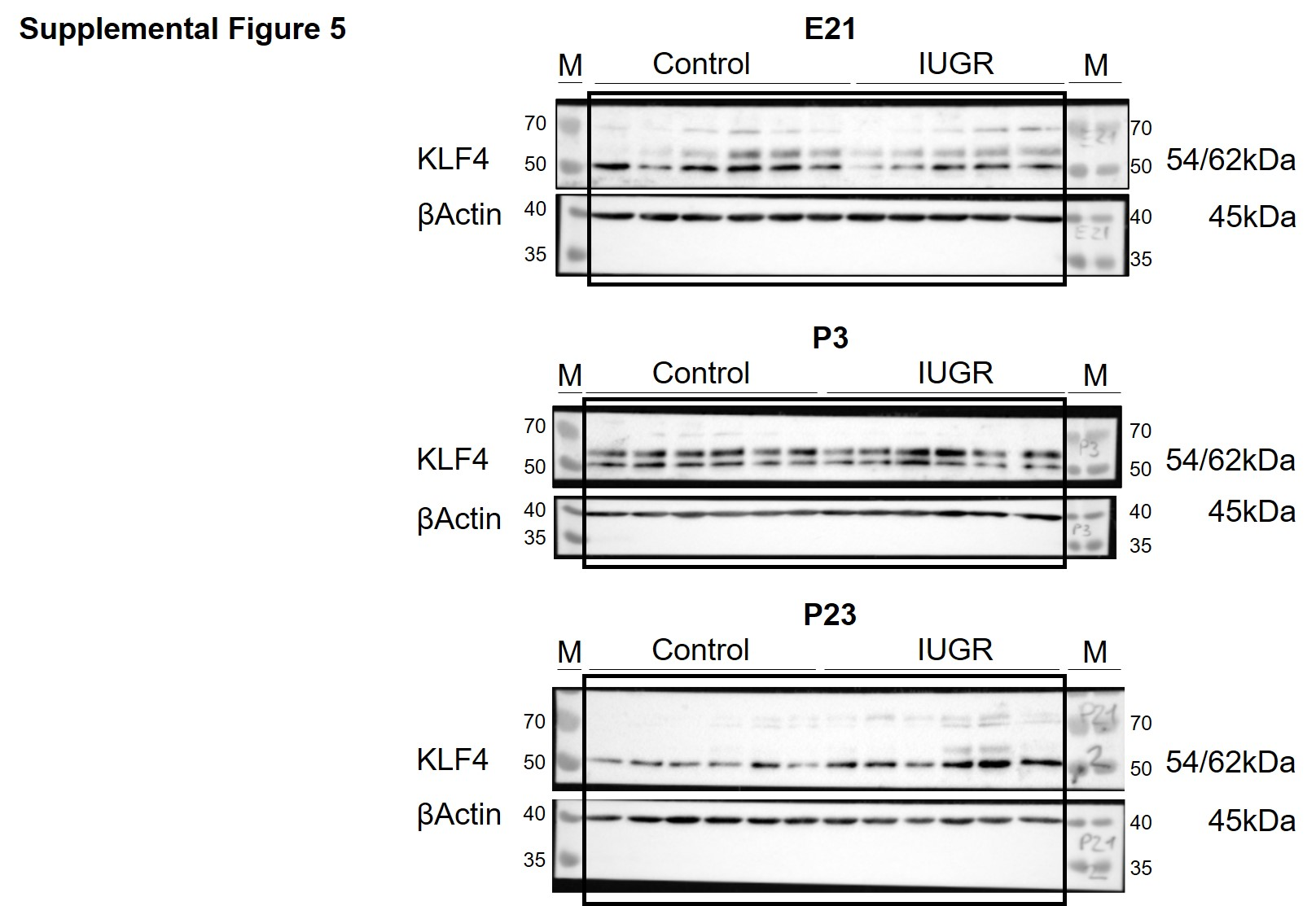


**Supplemental Figure 5.** Complete picture of immunoblots, as partly depicted in Figure 4D. Markers for molecular weight (M) are depicted on the left and right end of the blot, the molecular weight is represented in kDa. Protein expression of transcription factor Klf4 was measured (n=5-6/group), βActin served as a loading control.


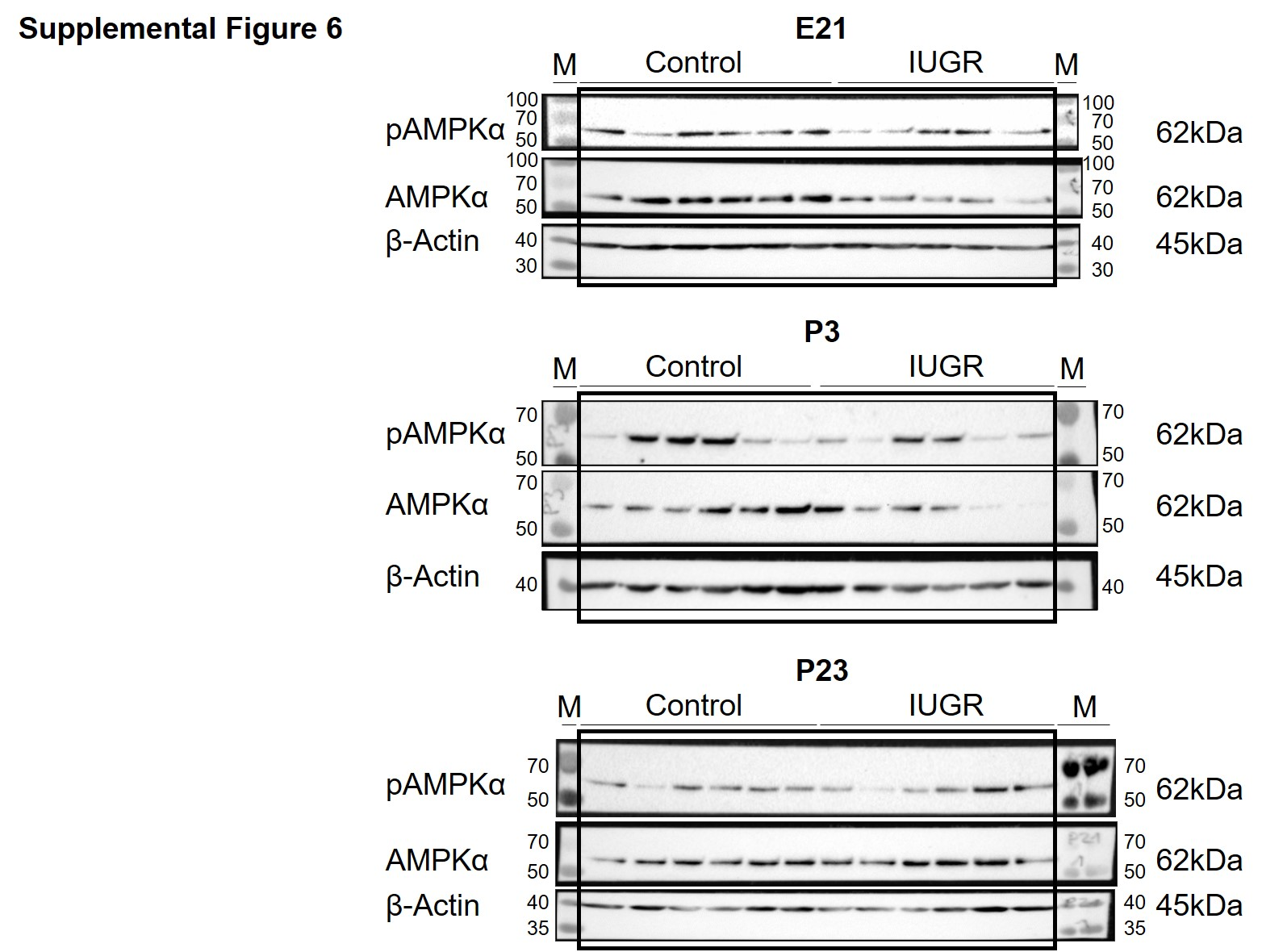


**Supplemental Figure 6.** Complete picture of immunoblots, as partly depicted in Figure 5A. Markers for molecular weight (M) are depicted on the left and right end of the blot, the molecular weight is represented in kDa. Immunoblots show the analysis of AMP-Activated protein kinase (AMPK)α-pathway in total lung homogenate on embryonic day (E21), postnatal day 3 (P3), and P23; total AMPKα and phosphorylated AMPKα (pAMPKα) were assessed; β-Actin served as loading control; (n=6/group).


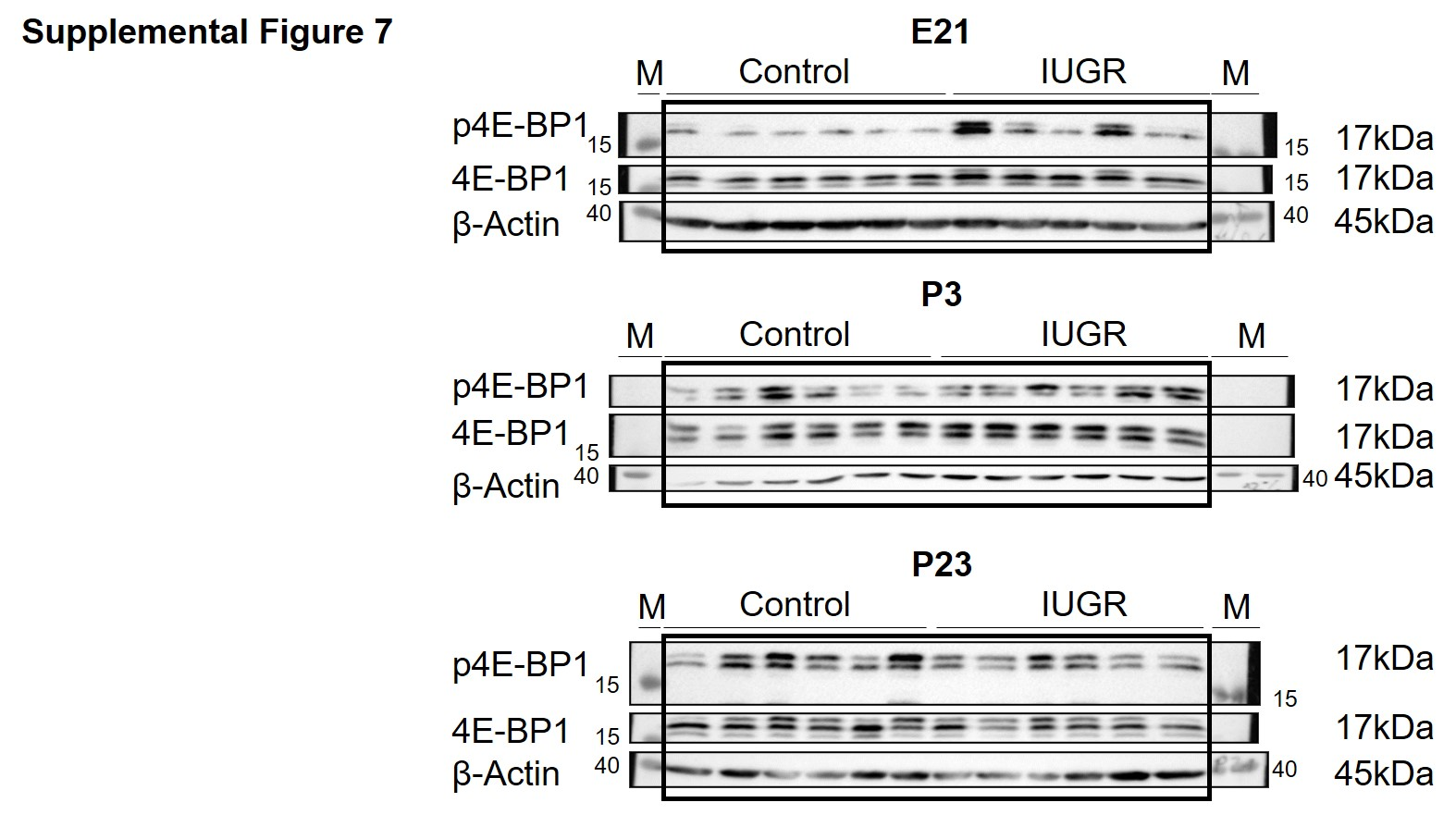


**Supplemental Figure 7.** Complete picture of immunoblots, as partly depicted in Figure 5B. Markers for molecular weight (M) are depicted on the left and right end of the blot, the molecular weight is represented in kDa. Total 4E-BP1 and phosphorylated 4E-BP1 (p4E-BP1) as a downstream effector of mTOR-pathway were assessed with immunoblot on E21, P3, and P23; β-Actin served as loading control; p4E-BP1 was related to β-Actin or to total 4E-BP1; (n=6/group).


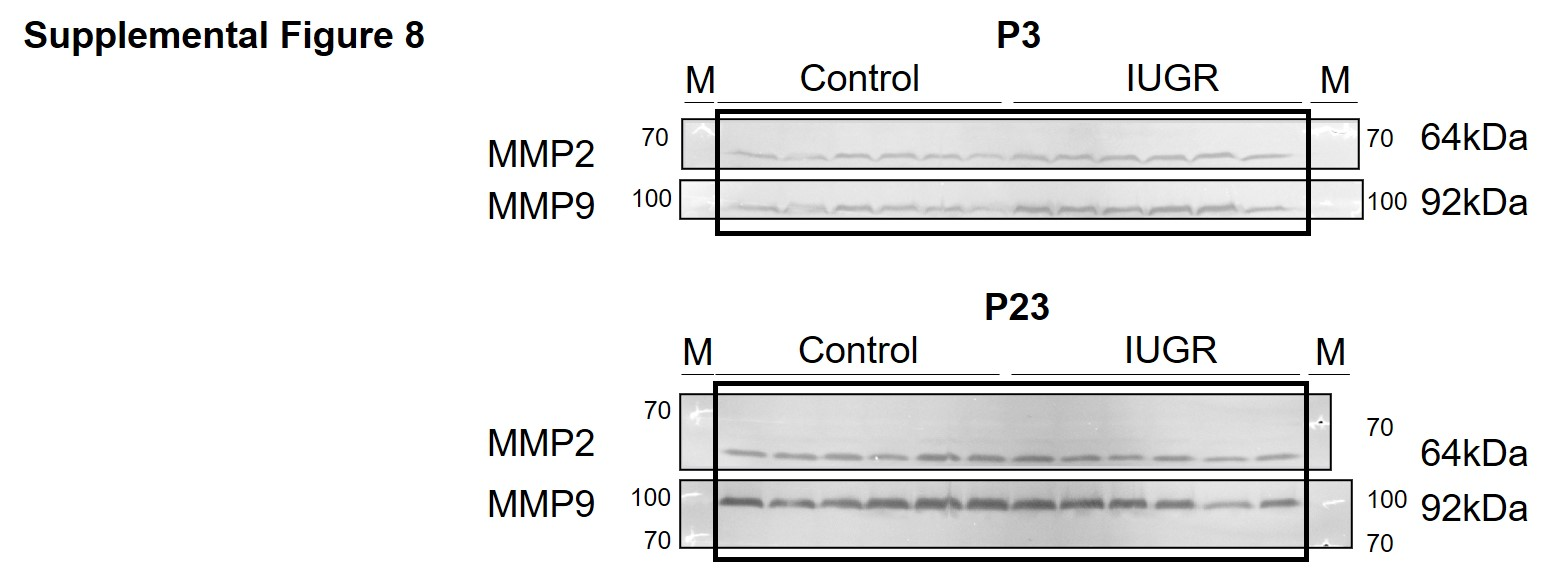


**Supplemental Figure 8.** Complete picture of Zymography as partly depicted in Figure 6, showing the analysis of the proteolytic activity of metalloprotease 2 and 9 (MMP2 at 64kDa, MMP9 at 92kDa) at P3 and P23. Markers for molecular weight (M) are depicted on the left and right end of the blot, the molecular weight is represented in kDa.

Supplemental Table 1

Variants included in the instrumental variable for Mendelian randomization analyses for own birth weight (a); offspring birth weight (b).

**1a**

| **SNP** | **Chromosome** | **Position** | **EA** | **OE** | **EAF** | **Beta** | **SE** | **P-value** | **Sample size** |
| --- | --- | --- | --- | --- | --- | --- | --- | --- | --- |
| **rs1482852** | **3** | **156798294** | **A** | **G** | **0,599** | **0,050** | **0,003** | **1,6E-82** | **298130** |
| **rs138715366** | **7** | **44246271** | **C** | **T** | **0,991** | **0,240** | **0,015** | **4,0E-61** | **284324** |
| **rs7968682** | **12** | **66371880** | **G** | **T** | **0,486** | **0,042** | **0,003** | **4,2E-60** | **298092** |
| **rs8756** | **12** | **66359752** | **C** | **A** | **0,487** | **0,041** | **0,003** | **2,4E-59** | **298139** |
| **rs17034876** | **2** | **46484310** | **T** | **C** | **0,700** | **0,042** | **0,003** | **3,1E-47** | **287749** |
| **rs35261542** | **6** | **20675792** | **C** | **A** | **0,733** | **0,041** | **0,003** | **2,8E-45** | **298124** |
| **rs11708067** | **3** | **123065778** | **G** | **A** | **0,238** | **0,041** | **0,003** | **1,6E-42** | **298128** |
| **rs4144829** | **4** | **17903654** | **C** | **T** | **0,267** | **0,036** | **0,003** | **4,3E-34** | **292713** |
| **rs2174633** | **4** | **17917781** | **A** | **C** | **0,270** | **0,035** | **0,003** | **7,1E-33** | **292712** |
| **rs10872678** | **6** | **152039964** | **T** | **C** | **0,724** | **0,032** | **0,003** | **9,8E-29** | **298136** |
| **rs7076938** | **10** | **115789375** | **T** | **C** | **0,735** | **0,032** | **0,003** | **2,1E-28** | **298136** |
| **rs7772579** | **6** | **152042502** | **A** | **C** | **0,721** | **0,031** | **0,003** | **6,4E-28** | **292718** |
| **rs28457693** | **9** | **98217348** | **G** | **A** | **0,109** | **0,044** | **0,004** | **9,9E-26** | **288037** |
| **rs1801253** | **10** | **115805056** | **C** | **G** | **0,727** | **0,031** | **0,003** | **1,4E-25** | **297700** |
| **rs222857** | **17** | **7164563** | **T** | **C** | **0,575** | **0,026** | **0,003** | **1,1E-24** | **298132** |
| **rs11698914** | **20** | **31327144** | **C** | **G** | **0,233** | **0,032** | **0,003** | **1,2E-24** | **292713** |
| **rs6845999** | **4** | **145565826** | **T** | **C** | **0,431** | **0,026** | **0,003** | **1,5E-24** | **298140** |
| **rs2131354** | **4** | **145599908** | **A** | **G** | **0,527** | **0,026** | **0,003** | **3,5E-24** | **292719** |
| **rs1112718** | **10** | **94479107** | **G** | **A** | **0,404** | **0,026** | **0,003** | **3,8E-23** | **298134** |
| **rs2428362** | **17** | **7180274** | **T** | **C** | **0,576** | **0,025** | **0,003** | **1,80E-22** | **292709** |
| **rs11042596** | **11** | **2118860** | **T** | **G** | **0,336** | **0,027** | **0,003** | **4,3E-22** | **292715** |
| **rs112139215** | **7** | **73034559** | **A** | **C** | **0,068** | **0,047** | **0,005** | **2,8E-20** | **295398** |
| **rs1012167** | **20** | **39159119** | **C** | **T** | **0,401** | **0,024** | **0,003** | **1,2E-19** | **292373** |
| **rs1480470** | **12** | **66412130** | **G** | **A** | **0,631** | **0,024** | **0,003** | **1,4E-19** | **292712** |
| **rs3184504** | **12** | **111884608** | **C** | **T** | **0,521** | **0,023** | **0,003** | **2,6E-19** | **296867** |
| **rs7402983** | **15** | **99193276** | **A** | **C** | **0,405** | **0,024** | **0,003** | **2,6E-19** | **292717** |
| **rs905938** | **1** | **154991389** | **C** | **T** | **0,262** | **0,026** | **0,003** | **2,8E-19** | **298135** |
| **rs9645500** | **10** | **70986723** | **G** | **T** | **0,694** | **0,024** | **0,003** | **1,8E-18** | **298136** |
| **rs5030938** | **10** | **70975916** | **T** | **C** | **0,686** | **0,024** | **0,003** | **1,2E-17** | **292718** |
| **rs13266210** | **8** | **41533514** | **A** | **G** | **0,786** | **0,027** | **0,003** | **1,5E-17** | **292718** |
| **rs2274224** | **10** | **96039597** | **C** | **G** | **0,434** | **0,021** | **0,003** | **9,8E-17** | **298132** |
| **rs56188432** | **2** | **158406865** | **G** | **A** | **0,002** | **0,258** | **0,031** | **1,4E-16** | **217397** |
| **rs55958435** | **15** | **96852638** | **A** | **G** | **0,748** | **0,025** | **0,003** | **1,6E-16** | **292710** |
| **rs2551347** | **2** | **23912401** | **T** | **C** | **0,749** | **0,024** | **0,003** | **1,9E-16** | **292714** |
| **rs10495563** | **2** | **9662210** | **A** | **G** | **0,664** | **0,022** | **0,003** | **2,1E-16** | **298133** |
| **rs75104038** | **6** | **34190104** | **A** | **G** | **0,060** | **0,045** | **0,006** | **4,3E-16** | **289515** |
| **rs10985827** | **9** | **125701608** | **G** | **T** | **0,141** | **0,030** | **0,004** | **6,1E-16** | **292715** |
| **rs6533183** | **4** | **106133184** | **C** | **T** | **0,352** | **0,022** | **0,003** | **6,8E-16** | **292715** |
| **rs854037** | **5** | **57091783** | **A** | **G** | **0,814** | **0,027** | **0,003** | **9,4E-16** | **292718** |
| **rs11893688** | **2** | **9695282** | **T** | **C** | **0,661** | **0,022** | **0,003** | **1,3E-15** | **292716** |
| **rs28505901** | **9** | **139241030** | **A** | **G** | **0,249** | **0,024** | **0,003** | **2,5E-15** | **286903** |
| **rs4444073** | **11** | **10331664** | **A** | **C** | **0,520** | **0,020** | **0,003** | **2,7E-15** | **298137** |
| **rs71486610** | **10** | **124134803** | **C** | **G** | **0,477** | **0,020** | **0,003** | **3,2E-15** | **292714** |
| **rs34776209** | **7** | **23513093** | **C** | **T** | **0,755** | **0,023** | **0,003** | **8,5E-15** | **292718** |
| **rs2418135** | **9** | **113901309** | **A** | **G** | **0,522** | **0,020** | **0,003** | **1,5E-14** | **292715** |
| **rs1411424** | **9** | **113892963** | **A** | **G** | **0,523** | **0,020** | **0,003** | **1,5E-14** | **292717** |
| **rs3933326** | **9** | **123633948** | **G** | **A** | **0,676** | **0,021** | **0,003** | **2,3E-14** | **292715** |
| **rs11096402** | **23** | **133827868** | **G** | **A** | **0,247** | **0,021** | **0,003** | **2,5E-14** | **267302** |
| **rs2292626** | **10** | **124186714** | **T** | **C** | **0,478** | **0,020** | **0,003** | **2,6E-14** | **292718** |
| **rs9379832** | **6** | **26186200** | **A** | **G** | **0,730** | **0,022** | **0,003** | **1,1E-13** | **291448** |
| **rs72656010** | **8** | **57122215** | **T** | **C** | **0,868** | **0,028** | **0,004** | **1,4E-13** | **292713** |
| **rs75034466** | **6** | **34199815** | **T** | **C** | **0,046** | **0,046** | **0,006** | **1,8E-13** | **289010** |
| **rs2934844** | **6** | **166142456** | **T** | **A** | **0,672** | **0,021** | **0,003** | **1,8E-13** | **292253** |
| **rs7223535** | **17** | **29211667** | **G** | **A** | **0,732** | **0,021** | **0,003** | **2,1E-13** | **292715** |
| **rs9348981** | **6** | **35687249** | **T** | **G** | **0,710** | **0,021** | **0,003** | **2,2E-13** | **292710** |
| **rs116807401** | **4** | **135121721** | **C** | **T** | **0,018** | **0,077** | **0,010** | **2,2E-13** | **265314** |
| **rs10935733** | **3** | **148622968** | **T** | **C** | **0,399** | **0,019** | **0,003** | **2,3E-13** | **292713** |
| **rs76895963** | **12** | **4384844** | **G** | **T** | **0,021** | **0,076** | **0,010** | **2,5E-13** | **278956** |
| **rs4719648** | **7** | **2756832** | **C** | **T** | **0,577** | **0,019** | **0,003** | **2,6E-13** | **292711** |
| **rs4932373** | **15** | **91429287** | **A** | **C** | **0,680** | **0,020** | **0,003** | **3,0E-13** | **295749** |
| **rs41311445** | **22** | **42070374** | **A** | **C** | **0,903** | **0,033** | **0,004** | **3,3E-13** | **289016** |
| **rs6930558** | **6** | **141878920** | **T** | **G** | **0,747** | **0,022** | **0,003** | **3,4E-13** | **292714** |
| **rs2306547** | **12** | **26877885** | **C** | **T** | **0,534** | **0,019** | **0,003** | **4,4E-13** | **292721** |
| **rs6040076** | **20** | **10658882** | **C** | **G** | **0,500** | **0,019** | **0,003** | **4,4E-13** | **292711** |
| **rs7819593** | **8** | **106115172** | **C** | **T** | **0,243** | **0,022** | **0,003** | **6,2E-13** | **292718** |
| **rs10283100** | **8** | **120596023** | **G** | **A** | **0,946** | **0,042** | **0,006** | **7,0E-13** | **291951** |
| **rs9549046** | **13** | **40647206** | **A** | **G** | **0,118** | **0,029** | **0,004** | **8,0E-13** | **291448** |
| **rs6575803** | **14** | **101257755** | **C** | **T** | **0,895** | **0,032** | **0,004** | **1,3E-12** | **284076** |
| **rs7183988** | **15** | **91428589** | **G** | **T** | **0,529** | **0,018** | **0,003** | **1,7E-12** | **294939** |
| **rs10181515** | **2** | **227019461** | **T** | **C** | **0,225** | **0,021** | **0,003** | **2,1E-12** | **298138** |
| **rs8106042** | **19** | **7161849** | **G** | **C** | **0,281** | **0,020** | **0,003** | **2,2E-12** | **291451** |
| **rs9851257** | **3** | **123125711** | **T** | **A** | **0,733** | **0,020** | **0,003** | **2,4E-12** | **298130** |
| **rs7886910** | **23** | **78630857** | **C** | **T** | **0,934** | **0,033** | **0,005** | **2,8E-12** | **266684** |
| **rs2647873** | **12** | **103081192** | **A** | **G** | **0,520** | **0,018** | **0,003** | **2,9E-12** | **292715** |
| **rs11055030** | **12** | **12878349** | **G** | **C** | **0,718** | **0,020** | **0,003** | **3,9E-12** | **292715** |
| **rs9318511** | **13** | **78601413** | **C** | **A** | **0,873** | **0,027** | **0,004** | **6,0E-12** | **292266** |
| **rs1547669** | **6** | **33775641** | **G** | **A** | **0,497** | **0,018** | **0,003** | **6,2E-12** | **289000** |
| **rs80278614** | **1** | **119412317** | **A** | **G** | **0,054** | **0,040** | **0,006** | **6,5E-12** | **292074** |
| **rs62496903** | **8** | **6446938** | **T** | **C** | **0,083** | **0,033** | **0,005** | **6,7E-12** | **290687** |
| **rs670523** | **1** | **155878732** | **G** | **A** | **0,669** | **0,019** | **0,003** | **7,6E-12** | **291451** |
| **rs667515** | **11** | **69449076** | **G** | **C** | **0,618** | **0,018** | **0,003** | **9,3E-12** | **292266** |
| **rs516246** | **19** | **49206172** | **C** | **T** | **0,506** | **0,018** | **0,003** | **9,3E-12** | **295749** |
| **rs73354194** | **17** | **79905947** | **C** | **T** | **0,025** | **0,061** | **0,009** | **1,0E-11** | **268519** |
| **rs7854962** | **9** | **96900505** | **C** | **G** | **0,785** | **0,022** | **0,003** | **1,0E-11** | **292711** |
| **rs2946179** | **5** | **157886627** | **C** | **T** | **0,734** | **0,020** | **0,003** | **1,1E-11** | **298129** |
| **rs732563** | **8** | **23345526** | **C** | **T** | **0,504** | **0,017** | **0,003** | **1,3E-11** | **292723** |
| **rs255773** | **19** | **54723546** | **C** | **T** | **0,536** | **0,018** | **0,003** | **1,3E-11** | **288702** |
| **rs6467157** | **7** | **127660763** | **T** | **C** | **0,713** | **0,020** | **0,003** | **1,5E-11** | **292717** |
| **rs76094073** | **6** | **109288036** | **G** | **C** | **0,121** | **0,027** | **0,004** | **1,6E-11** | **292719** |
| **rs6911621** | **6** | **35529025** | **T** | **C** | **0,344** | **0,018** | **0,003** | **1,6E-11** | **292722** |
| **rs7744700** | **6** | **53349401** | **T** | **A** | **0,711** | **0,020** | **0,003** | **1,6E-11** | **291448** |
| **rs28365970** | **5** | **67585723** | **C** | **A** | **0,741** | **0,020** | **0,003** | **1,7E-11** | **292712** |
| **rs2282978** | **7** | **92264410** | **C** | **T** | **0,326** | **0,018** | **0,003** | **1,7E-11** | **298140** |
| **rs73143584** | **20** | **62445702** | **A** | **G** | **0,110** | **0,029** | **0,004** | **1,8E-11** | **286584** |
| **rs78378222** | **17** | **7571752** | **G** | **T** | **0,013** | **0,079** | **0,012** | **1,8E-11** | **287415** |
| **rs13257363** | **8** | **142252580** | **G** | **A** | **0,591** | **0,018** | **0,003** | **2,0E-11** | **292711** |
| **rs9909342** | **17** | **25652275** | **A** | **G** | **0,381** | **0,018** | **0,003** | **2,2E-11** | **292713** |
| **rs13271368** | **8** | **126506140** | **C** | **T** | **0,761** | **0,020** | **0,003** | **2,3E-11** | **296867** |
| **rs3806315** | **1** | **214724668** | **A** | **G** | **0,591** | **0,018** | **0,003** | **2,8E-11** | **289070** |
| **rs10830963** | **11** | **92708710** | **G** | **C** | **0,277** | **0,019** | **0,003** | **2,8E-11** | **298126** |
| **rs9366778** | **6** | **31269173** | **G** | **A** | **0,627** | **0,018** | **0,003** | **2,9E-11** | **282578** |
| **rs12401656** | **1** | **43456767** | **G** | **A** | **0,865** | **0,025** | **0,004** | **3,4E-11** | **292712** |
| **rs4953353** | **2** | **46567276** | **G** | **T** | **0,632** | **0,018** | **0,003** | **3,5E-11** | **292721** |
| **rs72480273** | **1** | **161644871** | **C** | **A** | **0,182** | **0,023** | **0,003** | **4,0E-11** | **291667** |
| **rs220193** | **21** | **43581308** | **A** | **G** | **0,225** | **0,021** | **0,003** | **4,1E-11** | **292712** |
| **rs75844534** | **15** | **38667117** | **A** | **C** | **0,124** | **0,026** | **0,004** | **4,9E-11** | **292715** |
| **rs1323438** | **9** | **119115531** | **C** | **T** | **0,718** | **0,019** | **0,003** | **5,6E-11** | **292712** |
| **rs10221267** | **17** | **68464662** | **T** | **C** | **0,512** | **0,017** | **0,003** | **6,5E-11** | **296641** |
| **rs34217484** | **13** | **48854550** | **A** | **T** | **0,264** | **0,019** | **0,003** | **6,8E-11** | **287438** |
| **rs6426985** | **1** | **154813619** | **A** | **G** | **0,440** | **0,017** | **0,003** | **7,2E-11** | **292722** |
| **rs2229742** | **21** | **16339172** | **G** | **C** | **0,881** | **0,027** | **0,004** | **7,4E-11** | **297794** |
| **rs2779165** | **19** | **4915447** | **G** | **C** | **0,184** | **0,022** | **0,003** | **7,6E-11** | **291447** |
| **rs895964** | **12** | **26858066** | **G** | **A** | **0,532** | **0,017** | **0,003** | **7,9E-11** | **292722** |
| **rs1981627** | **5** | **133838180** | **G** | **A** | **0,585** | **0,017** | **0,003** | **8,4E-11** | **292716** |
| **rs34036147** | **8** | **38366249** | **T** | **C** | **0,688** | **0,018** | **0,003** | **8,4E-11** | **292711** |
| **rs11867479** | **17** | **68090207** | **T** | **C** | **0,353** | **0,017** | **0,003** | **1,1E-10** | **298138** |
| **rs4511593** | **17** | **7455536** | **T** | **C** | **0,650** | **0,017** | **0,003** | **1,1E-10** | **292717** |
| **rs41355649** | **19** | **33790556** | **G** | **A** | **0,934** | **0,034** | **0,005** | **1,2E-10** | **291155** |
| **rs10883846** | **10** | **104958244** | **C** | **T** | **0,615** | **0,017** | **0,003** | **1,3E-10** | **298138** |
| **rs144126567** | **1** | **161510516** | **C** | **G** | **0,925** | **0,033** | **0,005** | **1,6E-10** | **288421** |
| **rs116964396** | **8** | **41505849** | **A** | **C** | **0,035** | **0,045** | **0,007** | **2,0E-10** | **290436** |
| **rs10913200** | **1** | **176521655** | **G** | **A** | **0,972** | **0,051** | **0,008** | **2,0E-10** | **287089** |
| **rs339969** | **15** | **60883281** | **A** | **C** | **0,619** | **0,017** | **0,003** | **2,2E-10** | **292719** |
| **rs6026449** | **20** | **57272617** | **C** | **T** | **0,627** | **0,017** | **0,003** | **2,5E-10** | **292375** |
| **rs40434** | **16** | **55699525** | **G** | **A** | **0,391** | **0,017** | **0,003** | **3,0E-10** | **292714** |
| **rs11711420** | **3** | **183349010** | **T** | **G** | **0,747** | **0,019** | **0,003** | **3,2E-10** | **292710** |
| **rs7067170** | **23** | **68382836** | **G** | **A** | **0,760** | **0,017** | **0,003** | **3,8E-10** | **266751** |
| **rs2168443** | **3** | **46947087** | **T** | **A** | **0,379** | **0,017** | **0,003** | **3,9E-10** | **292713** |
| **rs3740360** | **10** | **96025491** | **C** | **A** | **0,109** | **0,026** | **0,004** | **4,0E-10** | **292719** |
| **rs11082304** | **18** | **20720973** | **T** | **G** | **0,508** | **0,016** | **0,003** | **4,2E-10** | **296792** |
| **rs67775399** | **1** | **161572353** | **C** | **T** | **0,219** | **0,021** | **0,003** | **4,4E-10** | **279004** |
| **rs61885091** | **11** | **69791952** | **A** | **G** | **0,169** | **0,023** | **0,004** | **4,8E-10** | **277677** |
| **rs6033062** | **20** | **11207419** | **A** | **T** | **0,460** | **0,016** | **0,003** | **5,2E-10** | **292717** |
| **rs134594** | **22** | **29468456** | **C** | **T** | **0,351** | **0,017** | **0,003** | **5,8E-10** | **290627** |
| **rs72681869** | **14** | **50655357** | **C** | **G** | **0,011** | **0,084** | **0,013** | **5,9E-10** | **259445** |
| **rs6569647** | **6** | **130337266** | **T** | **C** | **0,802** | **0,020** | **0,003** | **6,3E-10** | **292720** |
| **rs754868** | **2** | **43185532** | **G** | **A** | **0,419** | **0,016** | **0,003** | **6,7E-10** | **298139** |
| **rs2280235** | **2** | **191843830** | **G** | **A** | **0,259** | **0,018** | **0,003** | **6,9E-10** | **292718** |
| **rs1203876** | **20** | **22540915** | **C** | **A** | **0,046** | **0,038** | **0,006** | **9,4E-10** | **291539** |
| **rs2889874** | **20** | **33715777** | **G** | **T** | **0,452** | **0,016** | **0,003** | **9,4E-10** | **292712** |
| **rs6582623** | **12** | **46613394** | **C** | **T** | **0,869** | **0,024** | **0,004** | **1,1E-09** | **292715** |
| **rs61830764** | **1** | **212289976** | **A** | **G** | **0,377** | **0,017** | **0,003** | **1,1E-09** | **291445** |
| **rs10265057** | **7** | **47275737** | **G** | **A** | **0,092** | **0,027** | **0,004** | **1,3E-09** | **292446** |
| **rs1415701** | **6** | **130345835** | **G** | **A** | **0,736** | **0,018** | **0,003** | **1,4E-09** | **298129** |
| **rs2242116** | **3** | **46941116** | **A** | **G** | **0,391** | **0,015** | **0,003** | **1,5E-09** | **321211** |
| **rs147110934** | **19** | **55993436** | **G** | **T** | **0,975** | **0,052** | **0,009** | **1,6E-09** | **276061** |
| **rs234864** | **11** | **2857297** | **A** | **G** | **0,547** | **0,016** | **0,003** | **1,7E-09** | **296865** |
| **rs2306700** | **3** | **142123841** | **T** | **C** | **0,136** | **0,023** | **0,004** | **1,8E-09** | **290416** |
| **rs817329** | **20** | **62597694** | **T** | **G** | **0,406** | **0,016** | **0,003** | **2,3E-09** | **294502** |
| **rs59084784** | **7** | **22739562** | **A** | **C** | **0,323** | **0,017** | **0,003** | **2,4E-09** | **292716** |
| **rs708122** | **1** | **228216997** | **C** | **A** | **0,681** | **0,017** | **0,003** | **2,5E-09** | **292718** |
| **rs1129156** | **19** | **40719076** | **T** | **C** | **0,268** | **0,017** | **0,003** | **2,5E-09** | **292719** |
| **rs7285579** | **22** | **46441980** | **C** | **T** | **0,698** | **0,017** | **0,003** | **2,7E-09** | **290177** |
| **rs186606513** | **2** | **97482001** | **G** | **A** | **0,978** | **0,061** | **0,010** | **2,7E-09** | **280576** |
| **rs5030317** | **11** | **32410337** | **C** | **G** | **0,733** | **0,017** | **0,003** | **2,7E-09** | **292715** |
| **rs147957154** | **19** | **43431040** | **T** | **C** | **0,132** | **0,023** | **0,004** | **2,8E-09** | **269001** |
| **rs351930** | **5** | **52003397** | **T** | **A** | **0,801** | **0,019** | **0,003** | **2,9E-09** | **292714** |
| **rs6871635** | **5** | **133830395** | **G** | **A** | **0,566** | **0,016** | **0,003** | **3,0E-09** | **292716** |
| **rs11983722** | **7** | **46298647** | **A** | **T** | **0,938** | **0,032** | **0,005** | **3,1E-09** | **290622** |
| **rs9267812** | **6** | **32128394** | **T** | **C** | **0,133** | **0,023** | **0,004** | **3,1E-09** | **280156** |
| **rs753381** | **20** | **39797465** | **T** | **C** | **0,451** | **0,015** | **0,003** | **3,4E-09** | **297797** |
| **rs4350272** | **10** | **25056118** | **A** | **G** | **0,269** | **0,017** | **0,003** | **3,6E-09** | **298133** |
| **rs962554** | **6** | **142734204** | **T** | **C** | **0,715** | **0,017** | **0,003** | **3,8E-09** | **292717** |
| **rs1818782** | **5** | **39424628** | **C** | **A** | **0,637** | **0,016** | **0,003** | **4,2E-09** | **313072** |
| **rs13231367** | **7** | **127509070** | **G** | **A** | **0,714** | **0,017** | **0,003** | **4,4E-09** | **292714** |
| **rs2237467** | **7** | **50733316** | **A** | **G** | **0,221** | **0,018** | **0,003** | **5,3E-09** | **292710** |
| **rs151216** | **11** | **2680815** | **C** | **T** | **0,899** | **0,025** | **0,004** | **5,8E-09** | **298138** |
| **rs2045457** | **16** | **20046115** | **G** | **A** | **0,311** | **0,016** | **0,003** | **6,3E-09** | **292716** |
| **rs6925689** | **6** | **126865884** | **T** | **C** | **0,494** | **0,015** | **0,003** | **6,4E-09** | **292716** |
| **rs11085720** | **19** | **10317763** | **A** | **G** | **0,438** | **0,015** | **0,003** | **7,4E-09** | **315794** |
| **rs12153596** | **5** | **158410178** | **C** | **T** | **0,619** | **0,015** | **0,003** | **7,9E-09** | **292715** |
| **rs55836809** | **13** | **28502874** | **A** | **G** | **0,780** | **0,018** | **0,003** | **8,2E-09** | **291447** |
| **rs5750561** | **22** | **38595260** | **A** | **T** | **0,404** | **0,015** | **0,003** | **8,4E-09** | **290626** |
| **rs5742915** | **15** | **74336633** | **C** | **T** | **0,456** | **0,015** | **0,003** | **8,9E-09** | **296788** |
| **rs2663842** | **18** | **55449516** | **A** | **G** | **0,678** | **0,016** | **0,003** | **9,3E-09** | **287443** |
| **rs2262207** | **13** | **114136110** | **A** | **G** | **0,748** | **0,017** | **0,003** | **9,9E-09** | **290233** |
| **rs7205514** | **16** | **50271806** | **T** | **G** | **0,242** | **0,018** | **0,003** | **9,9E-09** | **287223** |
| **rs2967677** | **19** | **8789721** | **C** | **T** | **0,845** | **0,021** | **0,004** | **1,1E-08** | **284482** |
| **rs11704481** | **22** | **45732328** | **G** | **A** | **0,403** | **0,015** | **0,003** | **1,1E-08** | **290282** |
| **rs6568554** | **6** | **109290319** | **A** | **C** | **0,145** | **0,021** | **0,004** | **1,1E-08** | **292717** |
| **rs2967676** | **19** | **8789666** | **A** | **C** | **0,845** | **0,021** | **0,004** | **1,1E-08** | **284486** |
| **rs77553582** | **7** | **35299657** | **T** | **C** | **0,613** | **0,029** | **0,005** | **1,1E-08** | **75315** |
| **rs185262229** | **6** | **84105136** | **T** | **A** | **0,991** | **0,083** | **0,014** | **1,2E-08** | **278566** |
| **rs2395668** | **6** | **37105893** | **A** | **G** | **0,840** | **0,020** | **0,004** | **1,2E-08** | **292715** |
| **rs57414412** | **11** | **111769431** | **G** | **A** | **0,719** | **0,016** | **0,003** | **1,2E-08** | **314341** |
| **rs2189234** | **4** | **106075498** | **G** | **T** | **0,618** | **0,015** | **0,003** | **1,2E-08** | **292719** |
| **rs12623454** | **2** | **121326297** | **G** | **C** | **0,531** | **0,015** | **0,003** | **1,2E-08** | **292710** |
| **rs139429176** | **12** | **121632160** | **C** | **T** | **0,988** | **0,071** | **0,012** | **1,3E-08** | **280624** |
| **rs8038207** | **15** | **86316570** | **G** | **T** | **0,569** | **0,015** | **0,003** | **1,3E-08** | **292718** |
| **rs11646700** | **16** | **68421668** | **G** | **A** | **0,495** | **0,015** | **0,003** | **1,3E-08** | **292714** |
| **rs2807319** | **1** | **22554176** | **G** | **A** | **0,883** | **0,023** | **0,004** | **1,3E-08** | **292715** |
| **rs10173538** | **2** | **160569276** | **C** | **T** | **0,628** | **0,015** | **0,003** | **1,5E-08** | **292717** |
| **rs1937436** | **1** | **66441329** | **G** | **A** | **0,710** | **0,016** | **0,003** | **1,5E-08** | **292714** |
| **rs28681372** | **22** | **50351977** | **A** | **G** | **0,598** | **0,016** | **0,003** | **1,5E-08** | **284040** |
| **rs3965156** | **3** | **66484956** | **A** | **C** | **0,476** | **0,015** | **0,003** | **1,5E-08** | **292712** |
| **rs7525870** | **1** | **78269207** | **G** | **A** | **0,740** | **0,016** | **0,003** | **1,6E-08** | **298133** |
| **rs9508017** | **13** | **28934364** | **C** | **T** | **0,769** | **0,017** | **0,003** | **1,6E-08** | **292715** |
| **rs62562580** | **9** | **94252219** | **G** | **C** | **0,212** | **0,017** | **0,003** | **1,6E-08** | **315792** |
| **rs7075355** | **10** | **82208878** | **A** | **G** | **0,533** | **0,015** | **0,003** | **1,6E-08** | **292715** |
| **rs4809731** | **20** | **47495767** | **G** | **C** | **0,131** | **0,022** | **0,004** | **1,6E-08** | **292709** |
| **rs28544888** | **16** | **55741204** | **C** | **T** | **0,911** | **0,026** | **0,005** | **1,6E-08** | **292236** |
| **rs7541039** | **1** | **214176779** | **T** | **C** | **0,259** | **0,017** | **0,003** | **1,7E-08** | **292715** |
| **rs12802960** | **11** | **58174775** | **C** | **T** | **0,211** | **0,017** | **0,003** | **1,7E-08** | **315796** |
| **rs12656216** | **5** | **36160668** | **A** | **G** | **0,788** | **0,018** | **0,003** | **1,8E-08** | **292718** |
| **rs80019595** | **12** | **121417306** | **T** | **C** | **0,027** | **0,045** | **0,008** | **1,8E-08** | **295879** |
| **rs2608029** | **8** | **129170126** | **G** | **C** | **0,334** | **0,015** | **0,003** | **1,9E-08** | **292713** |
| **rs7563664** | **2** | **158344455** | **T** | **G** | **0,107** | **0,024** | **0,004** | **1,9E-08** | **292075** |
| **rs118106744** | **12** | **21936398** | **C** | **T** | **0,948** | **0,033** | **0,006** | **2,0E-08** | **289928** |
| **rs7102454** | **11** | **65594820** | **C** | **T** | **0,353** | **0,015** | **0,003** | **2,0E-08** | **292713** |
| **rs4867699** | **5** | **172748540** | **T** | **G** | **0,507** | **0,015** | **0,003** | **2,2E-08** | **308491** |
| **rs11545169** | **3** | **184020542** | **G** | **T** | **0,839** | **0,020** | **0,004** | **2,2E-08** | **292718** |
| **rs75518158** | **21** | **38393567** | **C** | **A** | **0,033** | **0,040** | **0,007** | **2,4E-08** | **302283** |
| **rs558443** | **10** | **90013195** | **A** | **C** | **0,775** | **0,017** | **0,003** | **2,5E-08** | **292268** |
| **rs2747503** | **6** | **15066121** | **C** | **T** | **0,663** | **0,016** | **0,003** | **2,7E-08** | **286757** |
| **rs11680809** | **2** | **113570809** | **A** | **C** | **0,567** | **0,015** | **0,003** | **2,8E-08** | **292720** |
| **rs2238464** | **16** | **2332577** | **T** | **C** | **0,321** | **0,015** | **0,003** | **2,8E-08** | **290626** |
| **rs7709066** | **5** | **77831071** | **C** | **T** | **0,455** | **0,014** | **0,003** | **2,9E-08** | **292717** |
| **rs12896104** | **14** | **74306014** | **G** | **A** | **0,674** | **0,015** | **0,003** | **3,1E-08** | **292711** |
| **rs1242516** | **17** | **17387079** | **C** | **T** | **0,860** | **0,021** | **0,004** | **3,2E-08** | **313076** |
| **rs12104672** | **2** | **109151173** | **T** | **G** | **0,574** | **0,014** | **0,003** | **3,3E-08** | **292711** |
| **rs9783782** | **16** | **88317285** | **G** | **T** | **0,312** | **0,016** | **0,003** | **3,4E-08** | **274031** |
| **rs7983505** | **13** | **33557173** | **T** | **A** | **0,166** | **0,018** | **0,003** | **3,5E-08** | **315793** |
| **rs1964859** | **20** | **607805** | **T** | **C** | **0,300** | **0,016** | **0,003** | **3,9E-08** | **291893** |
| **rs62023486** | **15** | **53070589** | **A** | **G** | **0,897** | **0,023** | **0,004** | **4,0E-08** | **310919** |
| **rs10147938** | **14** | **31885951** | **T** | **C** | **0,387** | **0,015** | **0,003** | **4,0E-08** | **292714** |
| **rs4794720** | **17** | **55392223** | **G** | **A** | **0,362** | **0,015** | **0,003** | **4,1E-08** | **292421** |
| **rs116276359** | **1** | **151821430** | **A** | **C** | **0,031** | **0,043** | **0,008** | **4,2E-08** | **285834** |
| **rs1967840** | **10** | **112026082** | **A** | **G** | **0,825** | **0,019** | **0,003** | **4,3E-08** | **292711** |
| **rs6958858** | **7** | **148966949** | **C** | **T** | **0,487** | **0,014** | **0,003** | **4,8E-08** | **287170** |
| **rs2755253** | **1** | **67470843** | **C** | **T** | **0,298** | **0,015** | **0,003** | **5,4E-08** | **292719** |

**1b**

| **SNP** | **Chromosome** | **Position** | **EA** | **OE** | **EAF** | **Beta** | **SE** | **P-value** | **Sample size** |
| --- | --- | --- | --- | --- | --- | --- | --- | --- | --- |
| rs10830963 | 11 | 92708710 | G | C | 0,279 | 0,045 | 0,003 | 9,1E-39 | 209954 |
| rs2946179 | 5 | 157886627 | C | T | 0,735 | 0,046 | 0,004 | 1,8E-37 | 197948 |
| rs3184504 | 12 | 111884608 | C | T | 0,518 | 0,037 | 0,003 | 1,4E-33 | 210260 |
| rs4679760 | 3 | 155855418 | G | C | 0,586 | 0,033 | 0,003 | 1,8E-25 | 197948 |
| rs2967676 | 19 | 8789666 | A | C | 0,842 | 0,044 | 0,004 | 2,2E-25 | 210262 |
| rs2967677 | 19 | 8789721 | C | T | 0,847 | 0,045 | 0,004 | 6,4E-25 | 197948 |
| rs2168101 | 11 | 8255408 | C | A | 0,691 | 0,033 | 0,003 | 2,9E-21 | 206024 |
| rs3740360 | 10 | 96025491 | C | A | 0,114 | 0,046 | 0,005 | 5,5E-21 | 210265 |
| rs180438 | 12 | 47187260 | G | A | 0,195 | 0,036 | 0,004 | 8,9E-21 | 210265 |
| rs7183988 | 15 | 91428589 | G | T | 0,526 | 0,029 | 0,003 | 1,4E-20 | 197947 |
| rs8756 | 12 | 66359752 | C | A | 0,486 | 0,028 | 0,003 | 1,1E-19 | 210262 |
| rs7968682 | 12 | 66371880 | G | T | 0,486 | 0,028 | 0,003 | 1,4E-19 | 210265 |
| rs17034876 | 2 | 46484310 | T | C | 0,696 | 0,030 | 0,003 | 1,4E-18 | 210261 |
| rs12909648 | 15 | 86224570 | G | A | 0,523 | 0,026 | 0,003 | 2,6E-18 | 210254 |
| rs75034466 | 6 | 34199815 | T | C | 0,048 | 0,062 | 0,007 | 1,6E-17 | 197947 |
| rs45446698 | 7 | 99332948 | G | T | 0,042 | 0,067 | 0,008 | 1,7E-17 | 197948 |
| rs4932373 | 15 | 91429287 | A | C | 0,675 | 0,028 | 0,003 | 1,7E-17 | 197948 |
| rs6871635 | 5 | 133830395 | G | A | 0,563 | 0,026 | 0,003 | 3,3E-17 | 210234 |
| rs9851257 | 3 | 123125711 | T | A | 0,744 | 0,030 | 0,004 | 7,2E-17 | 197947 |
| rs9379084 | 6 | 7231843 | G | A | 0,883 | 0,041 | 0,005 | 1,3E-16 | 208332 |
| rs75104038 | 6 | 34190104 | A | G | 0,061 | 0,054 | 0,007 | 2,1E-16 | 197947 |
| rs6911621 | 6 | 35529025 | T | C | 0,349 | 0,026 | 0,003 | 2,2E-16 | 210264 |
| rs1981627 | 5 | 133838180 | G | A | 0,581 | 0,025 | 0,003 | 2,6E-16 | 210238 |
| rs2131354 | 4 | 145599908 | A | G | 0,527 | 0,026 | 0,003 | 2,8E-16 | 197948 |
| rs2189234 | 4 | 106075498 | G | T | 0,616 | 0,026 | 0,003 | 2,9E-16 | 210262 |
| rs6533183 | 4 | 106133184 | C | T | 0,341 | 0,027 | 0,003 | 7,2E-16 | 197948 |
| rs17033114 | 12 | 103123339 | T | C | 0,934 | 0,054 | 0,007 | 1,1E-15 | 210248 |
| rs72760655 | 9 | 116916214 | C | A | 0,678 | 0,026 | 0,003 | 4,1E-15 | 197947 |
| rs560887 | 2 | 169763148 | C | T | 0,701 | 0,026 | 0,003 | 1,2E-14 | 210264 |
| rs6608539 | 23 | 115132834 | A | G | 0,468 | 0,024 | 0,003 | 2,5E-14 | 197093 |
| rs11051061 | 12 | 30914668 | A | G | 0,268 | 0,026 | 0,003 | 2,6E-14 | 207299 |
| rs1411424 | 9 | 113892963 | A | G | 0,523 | 0,023 | 0,003 | 2,9E-14 | 210255 |
| rs6845999 | 4 | 145565826 | T | C | 0,429 | 0,023 | 0,003 | 4,1E-14 | 210264 |
| rs2418135 | 9 | 113901309 | A | G | 0,519 | 0,023 | 0,003 | 6,8E-14 | 210248 |
| rs2971669 | 7 | 44231778 | T | C | 0,219 | 0,028 | 0,004 | 8,2E-14 | 210172 |
| rs6911024 | 6 | 31368451 | T | C | 0,902 | 0,038 | 0,005 | 1,9E-13 | 210233 |
| rs1482852 | 3 | 156798294 | A | G | 0,599 | 0,023 | 0,003 | 2,3E-13 | 210264 |
| rs138715366 | 7 | 44246271 | C | T | 0,991 | 0,124 | 0,017 | 2,5E-13 | 197947 |
| rs17367504 | 1 | 11862778 | G | A | 0,167 | 0,030 | 0,004 | 3,2E-13 | 210264 |
| rs34471628 | 5 | 172196752 | A | G | 0,962 | 0,059 | 0,008 | 3,7E-13 | 197948 |
| rs10509669 | 10 | 95969913 | A | T | 0,745 | 0,026 | 0,004 | 3,9E-13 | 210110 |
| rs10895278 | 11 | 102095335 | C | T | 0,340 | 0,023 | 0,003 | 6,7E-13 | 210263 |
| rs6995390 | 8 | 77611012 | T | A | 0,165 | 0,030 | 0,004 | 8,4E-13 | 210050 |
| rs2647873 | 12 | 103081192 | A | G | 0,519 | 0,022 | 0,003 | 1,2E-12 | 209613 |
| rs304001 | 19 | 56423668 | G | A | 0,394 | 0,022 | 0,003 | 2,6E-12 | 210264 |
| rs2174633 | 4 | 17917781 | A | C | 0,268 | 0,024 | 0,003 | 3,8E-12 | 210263 |
| rs6440006 | 3 | 141142691 | A | G | 0,448 | 0,021 | 0,003 | 4,3E-12 | 210249 |
| rs8038207 | 15 | 86316570 | G | T | 0,568 | 0,022 | 0,003 | 7,5E-12 | 197948 |
| rs3918226 | 7 | 150690176 | C | T | 0,919 | 0,040 | 0,006 | 9,0E-12 | 197948 |
| rs4952673 | 2 | 43423870 | A | G | 0,474 | 0,020 | 0,003 | 2,0E-11 | 210111 |
| rs3784789 | 15 | 75082552 | G | C | 0,674 | 0,022 | 0,003 | 2,2E-11 | 210261 |
| rs10734564 | 11 | 48160429 | A | G | 0,179 | 0,027 | 0,004 | 4,0E-11 | 197948 |
| rs2428362 | 17 | 7180274 | T | C | 0,571 | 0,021 | 0,003 | 5,1E-11 | 197093 |
| rs12461110 | 19 | 56320663 | A | G | 0,364 | 0,021 | 0,003 | 5,3E-11 | 209560 |
| rs4144829 | 4 | 17903654 | C | T | 0,262 | 0,023 | 0,004 | 6,7E-11 | 197947 |
| rs7903146 | 10 | 114758349 | T | C | 0,291 | 0,022 | 0,003 | 9,1E-11 | 210264 |
| rs9855896 | 3 | 14287150 | G | A | 0,222 | 0,023 | 0,004 | 2,4E-10 | 210257 |
| rs1724889 | 7 | 2741021 | G | A | 0,740 | 0,023 | 0,004 | 2,9E-10 | 197947 |
| rs5030938 | 10 | 70975916 | T | C | 0,687 | 0,020 | 0,003 | 4,6E-10 | 210264 |
| rs1801253 | 10 | 115805056 | C | G | 0,734 | 0,021 | 0,003 | 5,3E-10 | 210262 |
| rs7596521 | 2 | 46918665 | G | A | 0,271 | 0,021 | 0,003 | 5,5E-10 | 210265 |
| rs7772579 | 6 | 152042502 | A | C | 0,718 | 0,021 | 0,003 | 5,6E-10 | 210263 |
| rs1415701 | 6 | 130345835 | G | A | 0,729 | 0,022 | 0,003 | 6,4E-10 | 208908 |
| rs4579095 | 4 | 174726635 | G | A | 0,407 | 0,019 | 0,003 | 8,5E-10 | 210243 |
| rs1179494 | 2 | 36809496 | G | C | 0,672 | 0,020 | 0,003 | 9,1E-10 | 210204 |
| rs11641308 | 16 | 75312023 | T | C | 0,346 | 0,020 | 0,003 | 1,1E-09 | 197948 |
| rs11893688 | 2 | 9695282 | T | C | 0,666 | 0,020 | 0,003 | 1,1E-09 | 210263 |
| rs9645500 | 10 | 70986723 | G | T | 0,694 | 0,020 | 0,003 | 1,3E-09 | 210263 |
| rs12574749 | 11 | 32405355 | C | A | 0,720 | 0,021 | 0,003 | 1,3E-09 | 209490 |
| rs7808457 | 7 | 22798265 | A | T | 0,586 | 0,019 | 0,003 | 1,5E-09 | 210254 |
| rs6568554 | 6 | 109290319 | A | C | 0,144 | 0,027 | 0,004 | 1,7E-09 | 197948 |
| rs5030317 | 11 | 32410337 | C | G | 0,731 | 0,021 | 0,003 | 1,7E-09 | 210230 |
| rs562974282 | 10 | 104201070 | G | T | 0,002 | 0,212 | 0,035 | 1,8E-09 | 216895 |
| rs9912553 | 17 | 79959703 | G | C | 0,725 | 0,021 | 0,004 | 1,8E-09 | 197947 |
| rs10495563 | 2 | 9662210 | A | G | 0,668 | 0,019 | 0,003 | 1,8E-09 | 210265 |
| rs13231367 | 7 | 127509070 | G | A | 0,705 | 0,020 | 0,003 | 2,0E-09 | 210264 |
| rs222857 | 17 | 7164563 | T | C | 0,568 | 0,018 | 0,003 | 2,2E-09 | 209557 |
| rs10265133 | 7 | 45895604 | G | T | 0,858 | 0,027 | 0,005 | 2,3E-09 | 197947 |
| rs2928148 | 15 | 41401550 | A | G | 0,523 | 0,018 | 0,003 | 2,6E-09 | 210263 |
| rs181451002 | 20 | 32466219 | G | A | 0,979 | 0,063 | 0,011 | 3,0E-09 | 217750 |
| rs1533688 | 12 | 102772745 | C | T | 0,774 | 0,022 | 0,004 | 3,4E-09 | 197948 |
| rs10437653 | 11 | 46297631 | A | C | 0,500 | 0,017 | 0,003 | 3,5E-09 | 230068 |
| rs11778247 | 8 | 23403378 | G | A | 0,834 | 0,025 | 0,004 | 3,7E-09 | 197948 |
| rs28457693 | 9 | 98217348 | G | A | 0,106 | 0,030 | 0,005 | 3,7E-09 | 197948 |
| rs10872678 | 6 | 152039964 | T | C | 0,722 | 0,020 | 0,003 | 4,3E-09 | 210262 |
| rs12443252 | 15 | 91064690 | T | C | 0,550 | 0,018 | 0,003 | 4,8E-09 | 197948 |
| rs76895963 | 12 | 4384844 | G | T | 0,021 | 0,071 | 0,012 | 5,2E-09 | 197947 |
| rs3746448 | 20 | 33572979 | C | T | 0,836 | 0,024 | 0,004 | 7,0E-09 | 197948 |
| rs35549608 | 16 | 72227398 | C | T | 0,160 | 0,025 | 0,004 | 7,1E-09 | 197948 |
| rs7076938 | 10 | 115789375 | T | C | 0,732 | 0,020 | 0,003 | 7,3E-09 | 210265 |
| rs34717629 | 17 | 17610404 | A | G | 0,214 | 0,022 | 0,004 | 7,6E-09 | 197948 |
| rs34776209 | 7 | 23513093 | C | T | 0,752 | 0,021 | 0,004 | 7,9E-09 | 197948 |
| rs67775399 | 1 | 161572353 | C | T | 0,227 | 0,022 | 0,004 | 8,1E-09 | 197948 |
| rs2715026 | 4 | 120616805 | G | C | 0,598 | 0,018 | 0,003 | 9,0E-09 | 207381 |
| rs60573957 | 3 | 14884409 | T | C | 0,507 | 0,018 | 0,003 | 1,0E-08 | 197948 |
| rs2274224 | 10 | 96039597 | C | G | 0,433 | 0,018 | 0,003 | 1,0E-08 | 210042 |
| rs2242116 | 3 | 46941116 | A | G | 0,387 | 0,017 | 0,003 | 1,1E-08 | 229353 |
| rs76094073 | 6 | 109288036 | G | C | 0,121 | 0,027 | 0,005 | 1,2E-08 | 197947 |
| rs73226528 | 3 | 113398642 | C | T | 0,193 | 0,022 | 0,004 | 1,3E-08 | 217750 |
| rs2292626 | 10 | 124186714 | T | C | 0,473 | 0,017 | 0,003 | 1,4E-08 | 210265 |
| rs2118611 | 15 | 67401466 | T | C | 0,798 | 0,022 | 0,004 | 1,5E-08 | 210262 |
| rs41276588 | 1 | 118148384 | A | G | 0,287 | 0,019 | 0,003 | 1,7E-08 | 217750 |
| rs11957761 | 5 | 141058487 | G | A | 0,803 | 0,022 | 0,004 | 1,8E-08 | 209968 |
| rs787541 | 1 | 68051230 | C | G | 0,343 | 0,018 | 0,003 | 1,8E-08 | 229417 |
| rs809871 | 6 | 26256526 | C | G | 0,424 | 0,017 | 0,003 | 2,0E-08 | 210263 |
| rs139557015 | 2 | 182072326 | T | C | 0,965 | 0,049 | 0,009 | 2,0E-08 | 197948 |
| rs6467157 | 7 | 127660763 | T | C | 0,703 | 0,019 | 0,003 | 2,3E-08 | 210264 |
| rs4908404 | 1 | 28691734 | C | T | 0,470 | 0,017 | 0,003 | 2,7E-08 | 207380 |
| rs895964 | 12 | 26858066 | G | A | 0,526 | 0,017 | 0,003 | 2,7E-08 | 210259 |
| rs113510833 | 6 | 29671067 | T | C | 0,109 | 0,028 | 0,005 | 2,9E-08 | 217750 |
| rs4518515 | 6 | 142584429 | A | T | 0,436 | 0,018 | 0,003 | 3,0E-08 | 197948 |
| rs6602476 | 10 | 11122635 | A | C | 0,280 | 0,018 | 0,003 | 3,0E-08 | 230065 |
| rs2574727 | 3 | 11636508 | G | A | 0,946 | 0,039 | 0,007 | 3,0E-08 | 197948 |
| rs4809731 | 20 | 47495767 | G | C | 0,135 | 0,024 | 0,004 | 3,1E-08 | 230023 |
| rs3765041 | 11 | 64127230 | T | G | 0,259 | 0,019 | 0,003 | 3,2E-08 | 217749 |
| rs2307024 | 12 | 22005003 | T | G | 0,586 | 0,017 | 0,003 | 3,3E-08 | 230065 |
| rs6426985 | 1 | 154813619 | A | G | 0,442 | 0,017 | 0,003 | 3,5E-08 | 209613 |
| rs17315501 | 3 | 139029676 | G | A | 0,983 | 0,066 | 0,012 | 4,0E-08 | 217750 |
| rs10814916 | 9 | 4293150 | C | A | 0,513 | 0,016 | 0,003 | 4,3E-08 | 230060 |
| rs71486610 | 10 | 124134803 | C | G | 0,471 | 0,017 | 0,003 | 4,3E-08 | 197948 |
| rs636252 | 6 | 117157774 | T | C | 0,408 | 0,017 | 0,003 | 4,7E-08 | 210264 |
| rs12446550 | 16 | 28543381 | A | G | 0,419 | 0,017 | 0,003 | 4,7E-08 | 210256 |
| rs231350 | 11 | 2713649 | C | A | 0,645 | 0,018 | 0,003 | 4,8E-08 | 197947 |

Supplemental Table 2

Primer-sequences for qPCR analysis of angiogenic factors.

| **Taqman primer** | | |
| --- | --- | --- |
| *Pecam* (CD31) | forward | AATGTTGGGTGTGCAGAGAATG |
|  | reverse | CCCAAAAATGCAAGGATGTTC |
|  | taq | CCAGCCCACCCAGCCTGTCTTCTAAA |
| *Cdh5* (VE-Cadherin) | forward | GGTGCCAGTAAACACAAAGTTCAA |
|  | reverse | TCCCGATTAAACTGTCCATACTTG |
|  | taq | CATCAACAACCACGACAATACCGCCAAC |
| *vWF* | forward | TGGTCAGGATGTCCAGATTCC |
|  | reverse | CCCATAGCTGAGGCGTACAGA |
|  | taq | TCCTGCAAGGTGACCTCCGTATCCA |
| *Vegf* | forward | TGCCCACGTCGGAGAGCAACGT |
|  | reverse | TGATGAAGCCCTGGAGTGC |
|  | taq | TTGATCCGCATGATCTGCAT |
| *Flt1* (VEGF-R1) | forward | CAAATAAGCCCGCCGAGCCCC |
|  | reverse | CGGCAGACCAATACAATCCTAGA |
|  | taq | AAGAGTTTGACCACGGAGGAATC |
| *Flk1* (VEGF-R2) | forward | AGTGGCCCTGTCCCGTCCCC |
|  | reverse | GGACCACACTGCGCTCATC |
|  | taq | CTAAGCAGCACCTCTCGAGTTATTT |
| *Gapdh* | forward | CCAAGGAGTAAGAAACCCTGGACCACCC |
|  | reverse | TGTGAAGCTCATTTCCTGGTATGA |
|  | taq | CTCTCTTGCTCTCAGTATCCTTGCT |
| *Hprt* | forward | TGCAAGCTTGCTGGTGAAAAGGACCTCTC |
|  | reverse | GCAGTACAGCCCCAAAATGG |
|  | taq | TCATTATAGTCAAGGGCATATCCAAC |
| **SYBR primer** | | |
| *Bmpr1a* | forward | GCCTGTCTGTTCATCATTTCTCAT |
|  | reverse | GGTCCACGTCTGATTTCATACCA |
| *Bmpr1b* | forward | AAATGGAACTTGCTGCATAGCA |
|  | reverse | TGTTGGGTGGAATGTCAACCT |
| *Bmpr2* | forward | AACAAGGGTGCTGGTCTCAC |
|  | reverse | TGAGGGTGGGGTGGTAGTTA |
| *Apln* | forward | CTCTCCTTGACTGCCGTGTGT |
|  | reverse | TAGCGCATGTTGCCTTCTTCT |
| *Id1* | forward | GTTATCGACTACATCAGGGAC |
|  | reverse | GAACACATGCCGCCTC |
| *Klf4* | forward | GACCAGATGCAGTCGCAAGTC |
|  | reverse | AAGACCTTCTTCCCCTCTTTGG |
| *Eln* | forward | GAAAACCCCCGAAGCCCT |
|  | reverse | CCCCACCTTGATATCCCAGG |
| *Gapdh* | forward | CCCCTTCATTGACCTCAACTACA |
|  | reverse | GGATCTCGCTCCTGGAAGATG |

Supplemental Table 3

Primary and secondary antibodies for immunoblotting experiments.

| **Antibody** | **Abbreviation** | **Dilution** | **Manufacturer** | **Order number** | **Country** | **2nd Antibody** | **Dilution** |
| --- | --- | --- | --- | --- | --- | --- | --- |
| anti-von Willebrand Factor | vWF | 1:200 | DAKO | #A0082 | D | Rabbit | 1:200 |
| anti-vascular endothelial Cadherin | VE-Cadherin | 1:1000 | abcam | ab33168 | UK | Rabbit | 1:2000 |
| anti-cluster of differention 31 | CD31 | 1:5000 | Dianova | DIA-310 | DE | Rat | 1:5000 |
| anti- SMAD1 | SMAD1 | 1:1000 | cell signaling | #9743 | US | Rabbit | 1:2000 |
| anti-phosphorylated SMAD1 | pSMAD1/5/8 | 1:1000 | cell signaling | #9511 | US | Rabbit | 1:1000 |
| anti-extracellular-signal regulated kinases 1/2 | ERK1/2 | 1:3000 | cell signaling | #9102 | US | Rabbit | 1:4000 |
| anti-phosphorylated extracellular-signal regulated kinases 1/2 | pERK1/2 | 1:1000 | cell signaling | #4370 | US | Rabbit | 1:1000 |
| anti-5' AMP-activated protein kinase α | AMPKα | 1:1000 | cell signaling | #2603 | US | Rabbit | 1:2000 |
| anti-phosphorylated AMP-activated protein kinase α | pAMPKα | 1:1000 | cel signaling | #2535 | US | Rabbit | 1:1000 |
| anti-p70S6 | P70s6 | 1:500 | cell signaling | #2708 | US | Rabbit | 1:1000 |
| anti-phosphorylated p70S6 | pP70s6 | 1:2000 | cell signaling | #9234 | US | Rabbit | 1:500 |
| anti-4E-BP1 | 4E-BP1 | 1:2000 | cell signaling | #9644 | US | Rabbit | 1:2000 |
| anti-phosphrylated 4E-BP1 | p4E-BP1 | 1:1000 | cell signaling | #2855 | US | Rabbit | 1:2000 |
| anti-Krüppel-like factor 4 | KLF4 | 1:500 | cell signaling | #4038 | US | Rabbit | 1:1000 |
| anti-βActin | βActin | 1:5000 | cell signaling | #3700 | US | Mouse | 1:5000 |
| **Secondary Antibody** | | | **Manufacturer** | **Order number** | **Country** | | |
| Peroxidase-conjugated anti-mouse | | | cell signaling | #7077 | US | | |
| Peroxidase-conjugated anti-rabbit | | | cell signaling | #7074 | US | | |
| Peroxidase-conjugated anti-rat | | | cell signaling | #7076 | US | | |
